# Control of primary mouse cytomegalovirus infection in lung nodular inflammatory foci by cooperation of interferon-gamma expressing CD4 and CD8 T cells

**DOI:** 10.1371/journal.ppat.1007252

**Published:** 2018-08-28

**Authors:** Yvonne Lueder, Katrin Heller, Christiane Ritter, Kirsten A. Keyser, Karen Wagner, Xiaokun Liu, Martin Messerle, Felix R. Stahl, Stephan Halle, Reinhold Förster

**Affiliations:** 1 Institute of Immunology, Hannover Medical School, Hannover, Germany; 2 Institute of Virology, Hannover Medical School, Hannover, Germany; ETH Zurich, SWITZERLAND

## Abstract

Human cytomegalovirus (CMV) and mouse cytomegalovirus (MCMV) infection share many characteristics. Therefore infection of mice with MCMV is an important tool to understand immune responses and to design vaccines and therapies for patients at the risk of severe CMV disease. In this study, we investigated the immune response in the lungs following acute infection with MCMV. We used multi-color fluorescence microscopy to visualize single infected and immune cells in nodular inflammatory foci (NIFs) that formed around infected cells in the lungs. These NIFs consisted mainly of myeloid cells, T cells, and some NK cells. We found that the formation of NIFs was essential to reduce the number of infected cells in the lung tissue, showing that NIFs were sites of infection as well as sites of immune response. Comparing mice deficient for several leukocyte subsets, we identified T cells to be of prime importance for restricting MCMV infection in the lung. Moreover, T cells had to be present in NIFs in high numbers, and CD4 as well as CD8 T cells supported each other to efficiently control virus spread. Additionally, we investigated the effects of perforin and interferon-gamma (IFNγ) on the virus infection and found important roles for both mechanisms. NK cells and T cells were the major source for IFNγ in the lung and in *in vitro* assays we found that IFNγ had the potential to reduce plaque growth on primary lung stromal cells. Notably, the T cell-mediated control was shown to be perforin-independent but IFNγ-dependent. In total, this study systematically identifies crucial antiviral factors present in lung NIFs for early containment of a local MCMV infection at the single cell level.

## Author summary

Cytomegalovirus (CMV) is worldwide a highly prevalent β-herpesvirus. While the primary infection in healthy individuals does not cause disease, infection of immunocompromised patients can lead to multiple organ disease and can sometimes be lethal. CMV becomes latent and uses a series of mechanisms to circumvent its elimination by the immune system. Therefore, the establishment of therapies or vaccination strategies is a difficult endeavor. Murine CMV (MCMV) is a well-established model to study CMV infection in mice, since it targets many immune defense mechanisms in a similar manner as human CMV. Thus, studying immune responses of mice to MCMV can help to develop new therapeutic strategies for patients. In this study we focused on MCMV infection in adult mouse lungs and found that, in the immunocompetent host, immune cells infiltrate the lung tissue as nodular inflammatory foci that help to control the acute infection in one week. We identified specific lymphocyte subsets that are pivotal for efficient containment of the infection and showed that this process requires cooperation between CD4 and CD8 T cells. Furthermore, these cells need to secrete the multipotent cytokine IFNγ to successfully clear the lungs from infectious viruses.

## Introduction

The immune response against CMV infection in humans and murine cytomegalovirus (MCMV) in mice has been studied for decades [1]. Understanding immune-cell mediated control of CMV infection is of essential interest since immunocompromised patients are particularly susceptible to CMV-disease [2]. From earlier human and mouse studies, it is known that virus-specific T cell responses contribute to virus control in both species [3,4]. It is thought that both CD8 T cell-mediated killing of virus-infected cells and secretion of cytokines by CD8 and CD4 T cells contribute to protective immunity [4,5]. Since different lines of evidence indicate that T cells can control CMV infection, the adoptive transfer of HCMV-specific T cells has been used in clinical trials [6]. In patients that suffer from CMV reactivation or primary infection during periods of severe immunosuppression, the infusion of donor HCMV-specific T cells was found to improve the control of infection [7,8]. However, it remains unclear how CD8 T cells are able to control CMV infection, given that both HCMV and MCMV encode for proteins that efficiently down-modulate surface major histocompatibility complex class I (MHCI) on infected cells [9]. This immune evasion mechanism can prevent virus-specific T cells from recognizing their target cells and thus interferes with CD8 T cell-mediated killing [10]. Other immunoevasins interfere with the presentation of antigen via major histocompatibility complex class II (MHCII) molecules to avoid recognition by CD4 T cells [11,12] but they also downregulate ligands of natural killer (NK) cell receptors to avoid cytotoxic killing [13].

The immune response against CMV infection seems to be affected by the site of infection, since various cell populations and effector mechanisms differentially contribute to virus control in different organs [14–16]: While IFNγ secreting NK cells have a more pronounced role than CD8 T cells in controlling the infection in the liver [14,15], an early, perforin-dependent NK cell mediated, and a late CD8 T cell based mechanism control virus load in the spleen [14]. In salivary glands, IFNγ-secreting CD4 T cells are the major cell population crucial for viral reduction [16,17]. Although CMV pneumonitis is a common clinical problem, it is not entirely understood how the infection is controlled in lungs [18,19]. Moreover, it is still unclear which immune cells are locally present at the site of infection and how many cells of each type are needed to successfully control the infection in the affected tissue.

To simultaneously quantify the amount of infected cells and the ensuing cellular immune response at the primary site of infection, we have previously generated MCMV reporter mutants expressing the red fluorescent protein mCherry to directly observe single virus-infected cells by microscopy [20,21]. Following infection with these reporter viruses, histological sections of the infected organ reveal the number of virus-infected cells and simultaneously allow tracking of the immune response at the site of infection. Further, the MCMV reporter virus used in this study is deficient for m157. In C57BL/6 mice–but not in other mice, including wild mice–specific binding of the m157 protein to the NK-cell expressed Ly49H receptor leads to NK cell activation and fast elimination of infected cells [22]. Besides Ly49H, other specific NK cell receptors have been identified to be involved in the immune response during MCMV infection [23]. However, mice that do not express the activating NK cell receptor Ly49H are more susceptible to MCMV infection [24,25]. To investigate immunity against MCMV infection in C57BL/6 mice without an excessive NK cell response we here applied a m157-deficient reporter virus. Following infection of lungs in neonatal mice, we previously showed that MCMV infection can be precisely characterized, revealing the formation of dynamic nodular inflammatory foci (NIF) with accumulation of virus-infected and immune cells [26,27]. This inflammatory response is also found in humans after CMV infection and has been termed nodular inflammation due to the abundancy of myeloid cells and corresponding granuloma-like appearance [28]. Moreover, we observed how MCMV-specific CD8 T cells interact with infected cells to mediate antiviral immunity *in vivo* [10].

In the current study, we identified the types of immune cells that control acute MCMV infection in lung NIFs of adult mice. To systematically address the impact of different immune cells, we used several knock-out mice, as well as adoptive cell transfers, and cell depletion approaches. To clarify how leukocytes mediate antiviral immunity, we studied the main lymphocyte populations important for virus control in the lungs and the underlying mechanisms. For the lungs, we found that local inflammation with recruitment of immune cells was necessary to control infection. The formation of NIFs occurred independently of the presence of lymphoid cells. We confirmed an important role of T cell-secreted IFNγ, while perforin secretion by T cells was not essential for controlling MCMV infection in lung NIFs. However, in the absence of T cells we observed a NK cell-mediated antiviral effect that was independent from m157 recognition and conveyed by IFNγ and perforin. Furthermore, in adoptive cell transfer experiments we found that only the combination of CD8 with CD4 T cells was sufficient to control MCMV infection whereas either cell population on its own had only minor antiviral effects.

## Results

### Dynamics of MCMV infection in the lungs and local antiviral immunity

**Fig 1:**
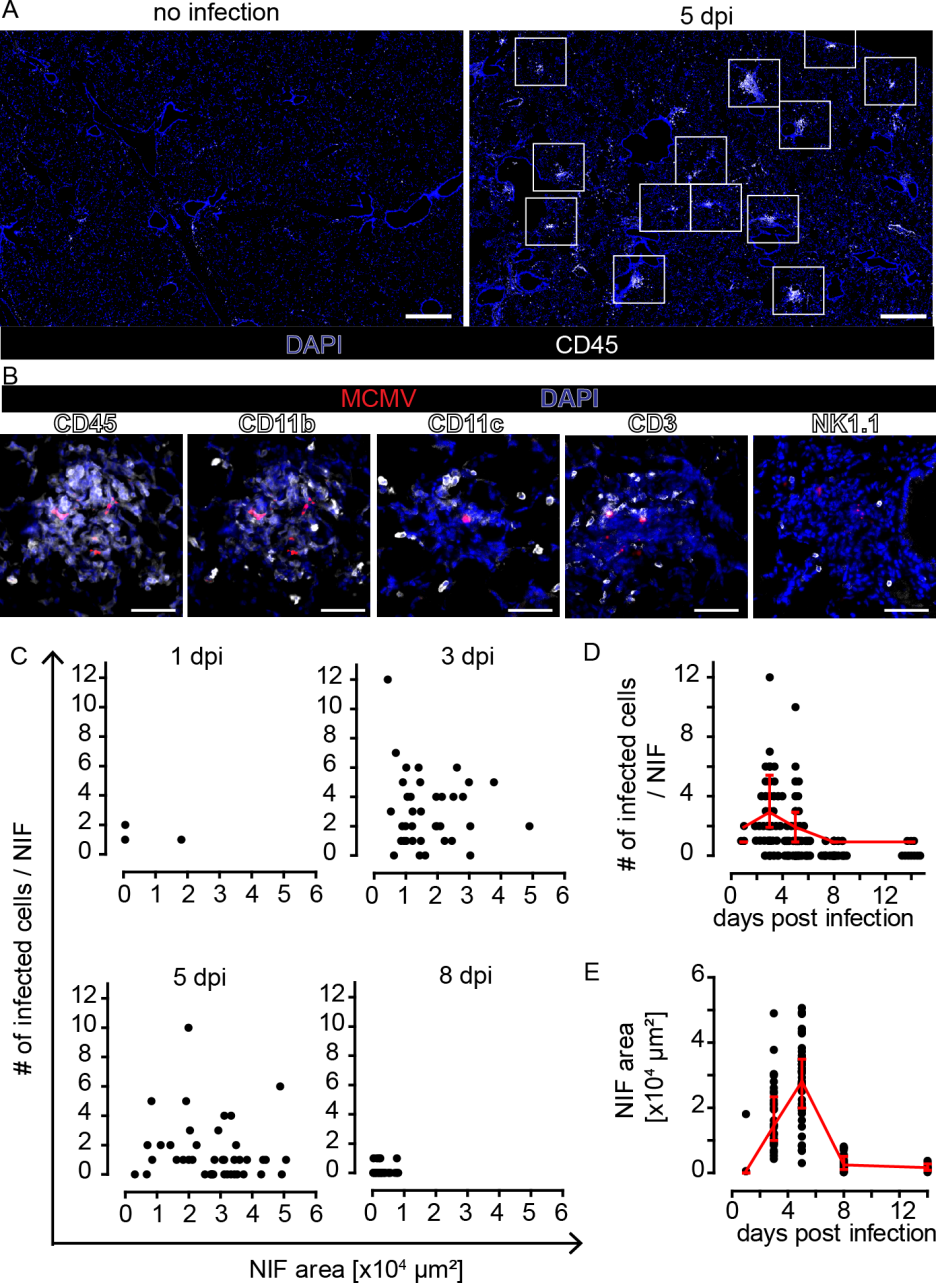
Infection of MCMV in the lungs is controlled around day 8 post infection in NIFs of wild-type mice. Animals were i.n. infected with 10^6^ PFU MCMV-3D. PFA-fixed lung cryosections were prepared from mice and analyzed 1, 3, 5, 8, and 14 dpi. **(A)** Overview of a lung lobe stained with an antibody against CD45 (white). Nuclear staining with DAPI (blue) revealing NIFs at different sites of the lung lobe at 5 dpi (right; non-infected mouse shown at the left). White squares indicate NIFs. Scale bars 500 μm. **(B)** NIFs at 5 dpi stained with antibodies as indicated (white), infected cells (red), and nuclear staining with DAPI (blue); scale bar 50 μm. Quantification of **(C)** number of infected cells per NIF section area. **(D)** Number of intact infected cells per NIF and **(E)** NIF size at different dpi as indicated. **(C-E)** 15–30 NIFs from 2–4 mice analyzed per time point, data pooled from 2 independent experiments per time point, dots represent NIFs, median + interquartile range (red).

To define the composition of immune cells in lung NIFs of adult immunocompetent mice, we intranasally infected 6–14 weeks old C57BL/6 mice with 10^6^ PFU of recombinant MCMV-3D [20]. At 5 days post infection (dpi), we found accumulations of CD45^+^ immune cells at multiple sites of the lungs (Fig 1A). In contrast, no inflammation could be observed in uninfected control animals (Fig 1A). The cellular infiltrates in lungs of MCMV-infected adult mice at 5 dpi localized in close proximity to MCMV-infected cells and consisted mainly of myeloid cells positive for CD11b and/or CD11c, as well as CD3^+^ T cells and NK1.1^+^ NK cells (Fig 1B). These findings demonstrate that adult mice develop NIFs around MCMV-infected cells in the lungs in a similar manner as previously described for infected neonatal mice [26]. Next, we analyzed the number of infected cells and NIF dynamics in the first two weeks following infection. We found an increasing number of MCMV-infected cells in NIFs within the first 3 dpi, whereas at 8 dpi only few mCherry^+^ cells remained (Fig 1C and 1D). In parallel, the average size of NIFs increased within the first 5 days and contracted until 8 dpi (Fig 1E). At day 8, NIFs were dissolving and only some mCherry^+^ remnants of infected cells were detected. Thus, in immunocompetent mice acute MCMV infection in the lungs was controlled during the first week of infection, most likely by cells that infiltrated the lung tissue and resided within NIFs at the site of infection.

### Gamma-irradiated mice do not form NIFs and fail to control local viral infection

**Fig 2:**
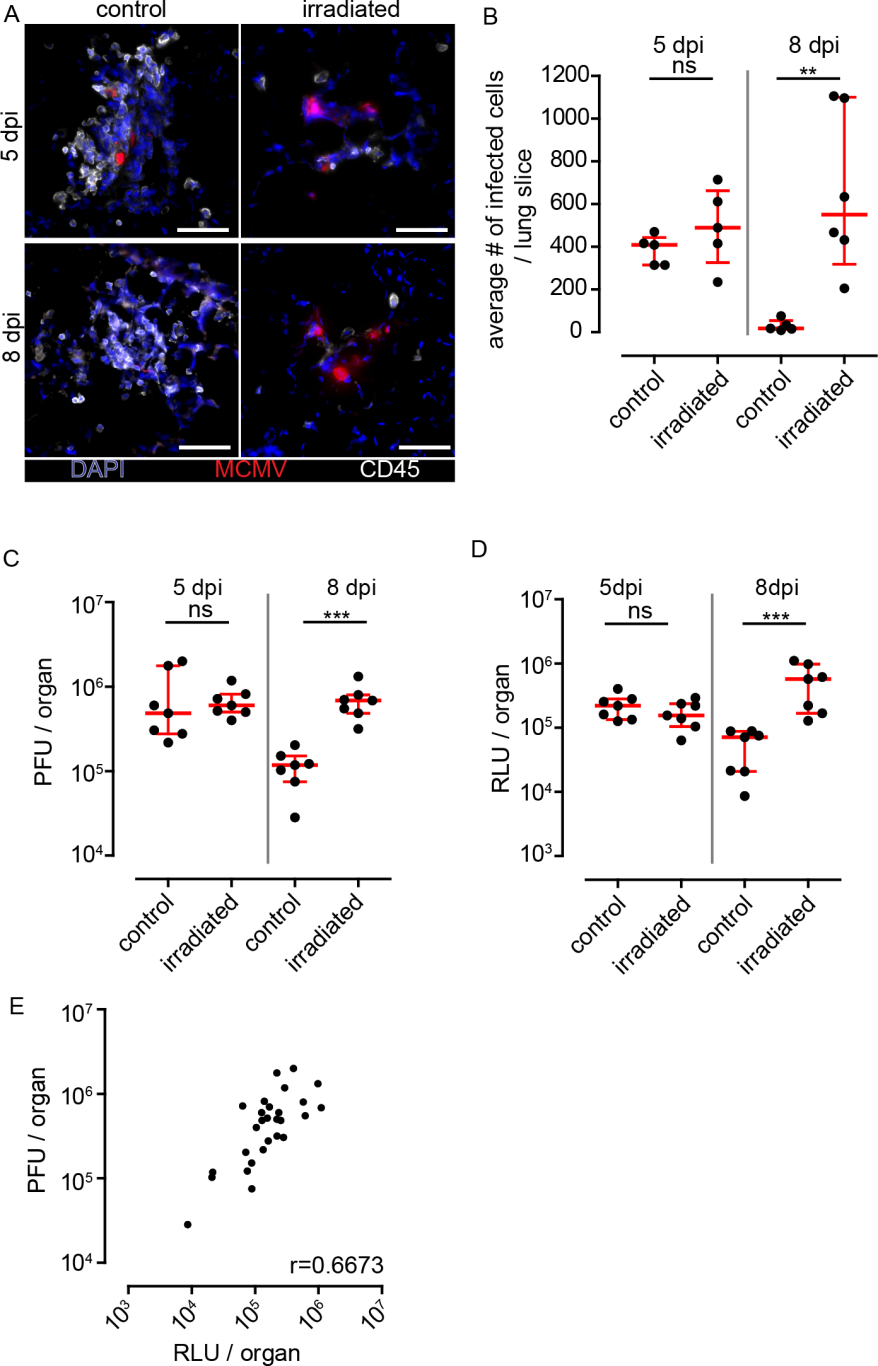
Absence of NIFs and increased infected cell counts in gamma-irradiated mice. Animals were either irradiated with 6 Gy or not (control) and infected one day post irradiation with 10^6^ PFU MCMV-3D i.n. **(A)** Immunofluorescence of NIFs or clusters of infected cells in irradiated (right panel) or control mice (left panel) 5 and 8 dpi stained with anti CD45 mAb (white), infected cells expressing mCherry (red) and nuclear staining with DAPI (blue); scale bar 50 μm. **(B)** Quantitative analysis of infected cells on histological sections of whole lungs, **(C)** analysis of virus titers in lungs, and **(D)** luciferase activity in lungs 5 and 8 dpi of irradiated and control mice. **(B)** Dots represent means of 3–4 lung slices analyzed per animal, **(C)** dots represent means of triplicates analyzed per organ, **(D)** dots represent mean of duplicates per organ; **(B-D)** median + interquartile range (red); data pooled from 2 independent experiments; Mann-Whitney test; **(E)** Correlation of virus titers and luciferase activity in lungs; pooled data of 5 and 8 dpi. Dots represent means per organ; r: Spearman coefficient. PFU, plaque forming units; RLU, relative light units; data pooled from 2 individual experiments.

To investigate whether hematopoietic cells present within NIFs mediate antiviral activity, we compared MCMV infection in gamma-irradiated versus non-irradiated mice. Following gamma-irradiation, mice showed reduced numbers of CD45^+^ cells in the peripheral blood, confirming interference with hematopoiesis. Five and eight days after MCMV infection of irradiated mice, we found no structures in the lungs that could be classified as NIFs (Fig 2A). Instead, we observed numerous clusters of morphologically intact MCMV-infected cells at both time points (Fig 2A). Only few apparently irradiation-resistant hematopoietic cells were scattered through the lung tissue. Because of the absence of NIFs in irradiated mice, we quantified by histology the number of infected cells per lung slice and measured in a separate experiment virus titers and Gaussia luciferase activities (Fig 2B–2E). At 5 dpi, we found the number of intact infected cells, Gaussia luciferase activity, and virus titers to be comparable in irradiated and control animals. In contrast, at 8 dpi, control mice showed a reduction in infected cell numbers, luciferase activities, and virus titers, whereas in irradiated mice virus loads in the lungs had slightly increased compared to 5 dpi (Fig 2B–2D). In general, we found a correlation between viral titers and luciferase activity in lungs as well as salivary glands (Fig 2E, S1C Fig). Analysis of salivary glands showed a comparable infection level in irradiated and control mice at 5 dpi (S1A and S1B Fig). However, virus loads were increased in both conditions at 8 dpi indicating that the virus spreads slowly towards salivary glands (S1A and S1B Fig), confirming previous reports on infection dynamics in salivary glands [26,29]. Furthermore, virus loads in irradiated mice were even higher at 8 dpi than control mice, suggesting a role for immune cells to reduce virus dissemination from the lungs into salivary glands.

Taken together, the disappearance of MCMV-infected cells in lungs of immunocompetent mice was most likely due to antiviral effects executed by NIF-resident immune cells.

### Control of virus spread in NIFs depends primarily on local T cells, while NIF formation is lymphocyte independent

**Fig 3:**
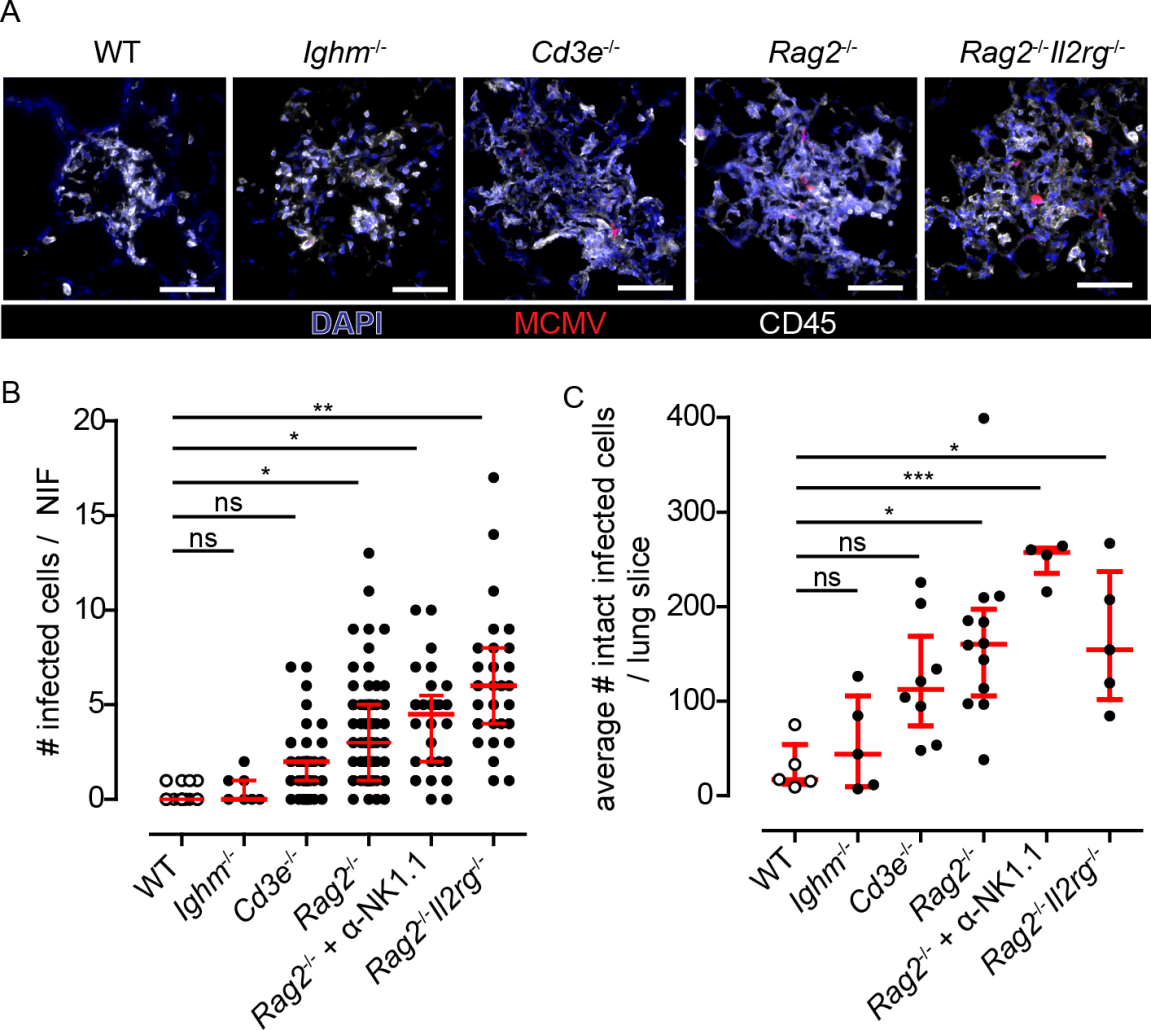
Role of different lymphocyte subsets in the formation of NIFs and in the control of viral replication in the lung. Various mouse strains deficient for different cell populations were infected with 10^6^ PFU MCMV-3D, i.e. *Ighm*^-/-^, *Cd3e*^-/-^, *Rag2*^-/-^, and *Rag2*^-/-^*Il2rg*^-/-^ mice and analyzed at 8dpi. **(A)** Immunofluorescence of NIFs in different mouse strains stained as indicated with an antibody against CD45 (white) and a nuclear staining with DAPI (blue); infected cells expressing mCherry (red); Scale bar 50 μm. **(B+C)** Quantification of the number of infected cells counted **(B)** per NIF or **(C)** per whole lung slice. **(B)** Dots represent NIFs; Mann-Whitney test compared to WT data performed with mean values of individual animals. **(C)** Dots represent means of 4 lung slices analyzed per animal. **(B+C)** open circles (WT) are identical to that shown in Fig 1C and 1D and Fig 2B; median + interquartile range (red); Mann-Whitney test compared to WT data performed with mean values of individual animals; pooled data from 2–3 independent experiments.

Next, we asked whether NIF formation occurs in the absence of defined subpopulations of immune cells and whether virus spread can be controlled under such conditions. To screen for the contribution of different types of lymphoid cells to NIF formation and antiviral control, we used mice deficient for B cells (*Ighm*^-/-^), T cells (*Cd3e*^-/-^), B and T cells (*Rag2*^-/-^), and mice lacking the entire lymphoid compartment (*Rag2*^-/-^*Il2rg*^-/-^). Following intranasal infection, we found typical NIFs in all these immunodeficient mouse strains (Fig 3A). At 8 dpi, NIFs in *Ighm*^-/-^ mice were already dissolving and negative for intact mCherry^+^ cells, thus being comparable to observations made in immunocompetent mice (Fig 3A and 3B). In contrast, in T cell-deficient *Cd3e*^-/-^ mice as well as T and B cell-deficient *Rag2*^-/-^ mice NIFs were still present and contained intact mCherry^+^ cells (Fig 3A and 3B and S2 Fig). These animals showed a small but consistently higher number of infected cells per NIF than observed in wild-type mice (Fig 3B), while a robust elevation of the total number of infected cells could be observed (Fig 3C). Mice deficient for T, B, and NK cells (*Rag2*^-/-^*Il2rg*^-/-^ mice) or *Rag2*^-/-^ mice depleted for NK cells showed the highest average number of virus-infected cells per NIF, suggesting that the different types of lymphocytes cooperate to limit virus replication inside NIFs (Fig 3B). Notably, we found no difference in the average number of infected cells per lung slice between *Rag2*^-/-^*Il2rg*^-/-^ and *Rag2*^-/-^ mice, while NK cell-depleted *Rag2*^-/-^ mice showed elevated numbers of infected cells (Fig 3C). To further address the role of NK cells in the presence of T and B cells we next depleted NK cells in WT mice. For these experiments we chose to analyze the lungs at 5 dpi, since the NK cell-mediated response is faster than T cells and at 8 dpi the infection is already controlled in WT mice. In NK cell-depleted mice we could detect slightly increased numbers of infected cells per NIF but not per lung slice (S3 Fig). Together, these data suggest that NIF formation occurs independently of the presence of lymphocytes and control of viral infection in NIFs primarily relied on T cells. Further, independent of m157, NK cells can reduce the infection in the absence of T cells and may have the potential to counteract the infection in the presence of T cells.

The MCMV-encoded chemokine 2 (MCK2) was reported to increase virulence of the virus [27,30–32] and may interfere with T cell responses [33,34]. To address whether the presence of MCK2 affects T cell-mediated control of lung MCMV infection we infected WT and *Rag2*^-/-^ mice with MCMV-3DR. In contrast to the MCMV-3D recombinant the insertion of a missing base pair in the *Mck2* ORF of the MCMV-3DR recombinant allows for accurate translation of the whole MCK2 protein [30]. Similarly to the MCMV-3D virus, the number of infected cells per NIF in *Rag2*^*-/-*^ mice was increased compared to WT mice infected with MCMV-3DR at 8 dpi (S4A Fig). However, MCMV-3DR infected alveolar macrophages leading to a higher total number of infected cells per lung as compared to animals infected with MCMV-3D –which shows no tropism to alveolar macrophages (S4B Fig) [27]. Thus, the T cell-mediated control of MCMV infection in NIFs seems to be independent of the presence of the full MCK2 protein.

### Control of local MCMV infection depends on the number of T cells present in NIFs

**Fig 4:**
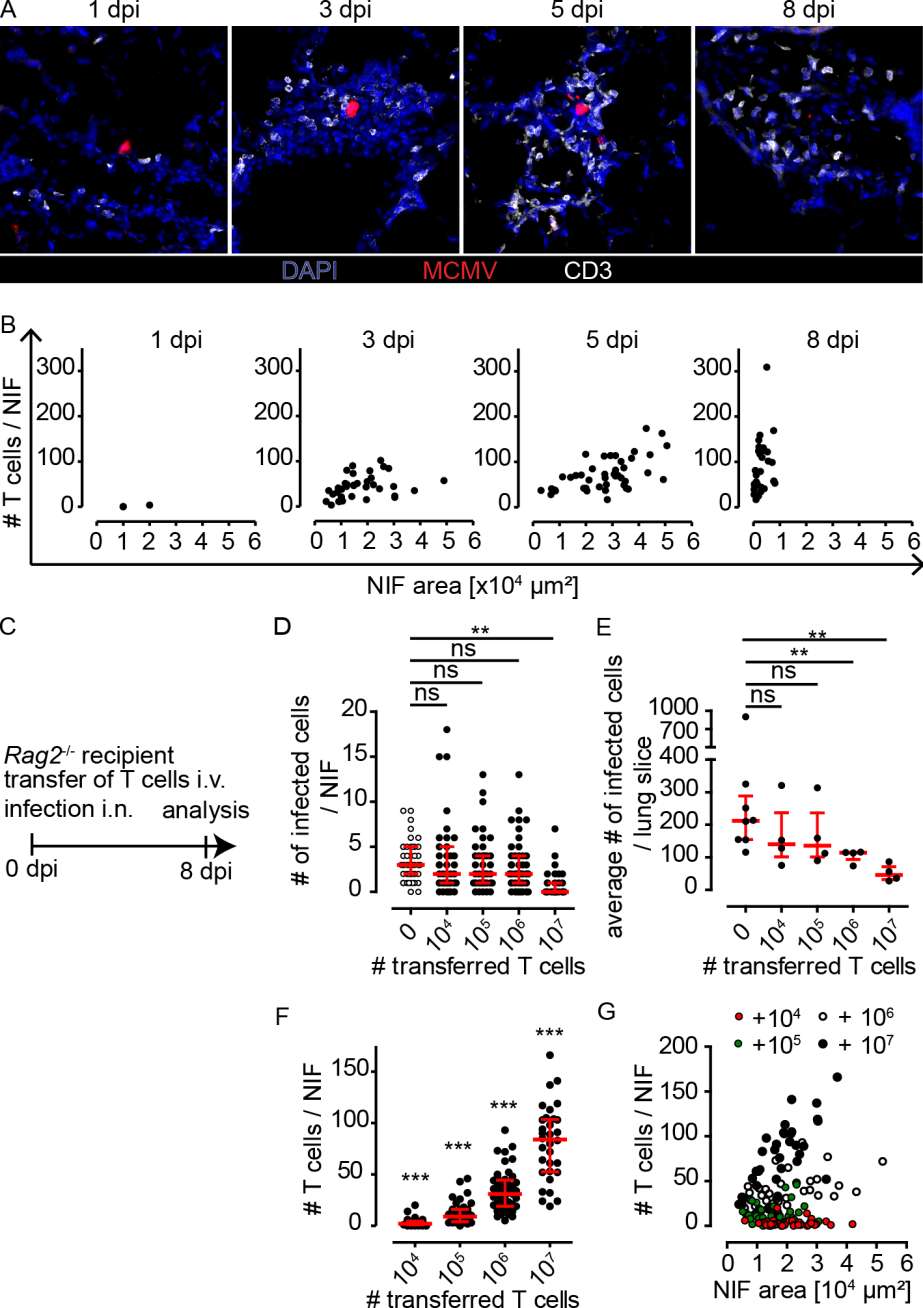
Number of T cells present in NIFs increases over time and can control infection if present in high numbers. **(A+B)** Animals were i.n. infected with 10^6^ PFU MCMV-3D. **(A)** Histology of representative NIFs stained for CD3 (white) and DAPI (blue) and **(B)** quantification of the number of T cells present in NIFs per area of individual NIFs at 1, 3, 5, and 8 dpi as indicated. Infected mCherry^+^ cells shown in red; scale bar 50 μm; dots represent NIFs. **(C)** Various numbers of wild-type T cells were adoptively transferred into *Rag2*^-/-^ mice infected with 10^6^ PFU of MCMV-3D. Lung histology was analyzed 8 dpi. **(D+E)** Analysis of intact infected cells per **(D)** NIF or **(E)** lung slice. For comparison data from infected *Rag2*^-/-^ mice without T cell transfer are shown; **(F)** Quantification of T cells present in NIFs. **(G)** Analysis of T cell densities per NIF. **(D+F+G)** dots represent NIF from 3–4 animals of 2 independent experiments, **(E)** dots represent animals (mean of 4 lung slices per animal). **(D-F)** median + interquartile range (red). **(D+E)** Mann-Whitney-test compared to *Rag2*^-/-^ without T cell transfer performed with mean values of individual animals. **(F)** Wilcoxon Signed Rank Test compared to 0 (number of T cells in *Rag2*^-/-^ mice without T cells). **(D-G)** Pooled data from 2 independent experiments.

We next studied the dynamics of the number of lymphocytes present in NIFs of MCMV-infected wild-type mice. Histological analysis at 1 dpi revealed hardly any lymphocytes residing in close proximity to infected cells, whereas at 3 dpi and 5 dpi a substantial number of T cells is present in NIFs. The number of T cells per NIF is even further increased at 8 dpi, when most of the infected cells already disappeared. In contrast, the number of NK cells present in NIFs peaked at 3 dpi and declined until 5 dpi (Fig 4A and 4B and S5 Fig). These data indicate that NK cells populate NIFs early after infection, while T cells might mediate their antiviral effects between 3 and 8 dpi.

To determine whether the T cell-mediated antiviral response is dose-dependent, we varied the number of T cells available in the lungs of infected mice. To this end, we adoptively transferred increasing numbers of purified polyclonal T cells isolated from untreated wild-type mice into MCMV-infected *Rag2*^-/-^ recipients (Fig 4C). At day 8 post infection and transfer of 10^6^ or less T cells, many NIFs still contained virus-infected cells (Fig 4D). In contrast, the transfer of 10^7^ T cells resulted in a reduced number of infected cells per NIF (Fig 4D). We observed a similar effect when analyzing the whole lung slice for the average number of infected cells (Fig 4E). Simultaneously, we analyzed the number of T cells present in NIFs. Only few T cells were detected in mice receiving 10^4^ or 10^5^ T cells and a robust increase in the number of T cells was observed after transfer of 10^6^ T cells (Fig 4F). However, following the adoptive transfer of 10^7^ T cells into *Rag2*^-/-^ recipients, we found T cell densities comparable to those in NIFs of wild-type mice (approximately 100 T cells per NIF; Fig 4G). Interestingly, FACS analysis of infected lungs revealed that a substantial amount of CD8 T cells is specific for MCMV (S6 Fig). These data indicate that a certain T cell density is needed to sufficiently contain the MCMV infection. In summary, we found that the number of T cells per NIF increased over time in wild-type mice and that adoptive transfer of 10^7^ naïve T cells into *Rag2*^-/-^ mice was necessary to accumulate enough T cells in NIFs to control the infection.

### Molecular mechanisms of T cell- and NK cell-mediated antiviral immunity in NIFs

**Fig 5:**
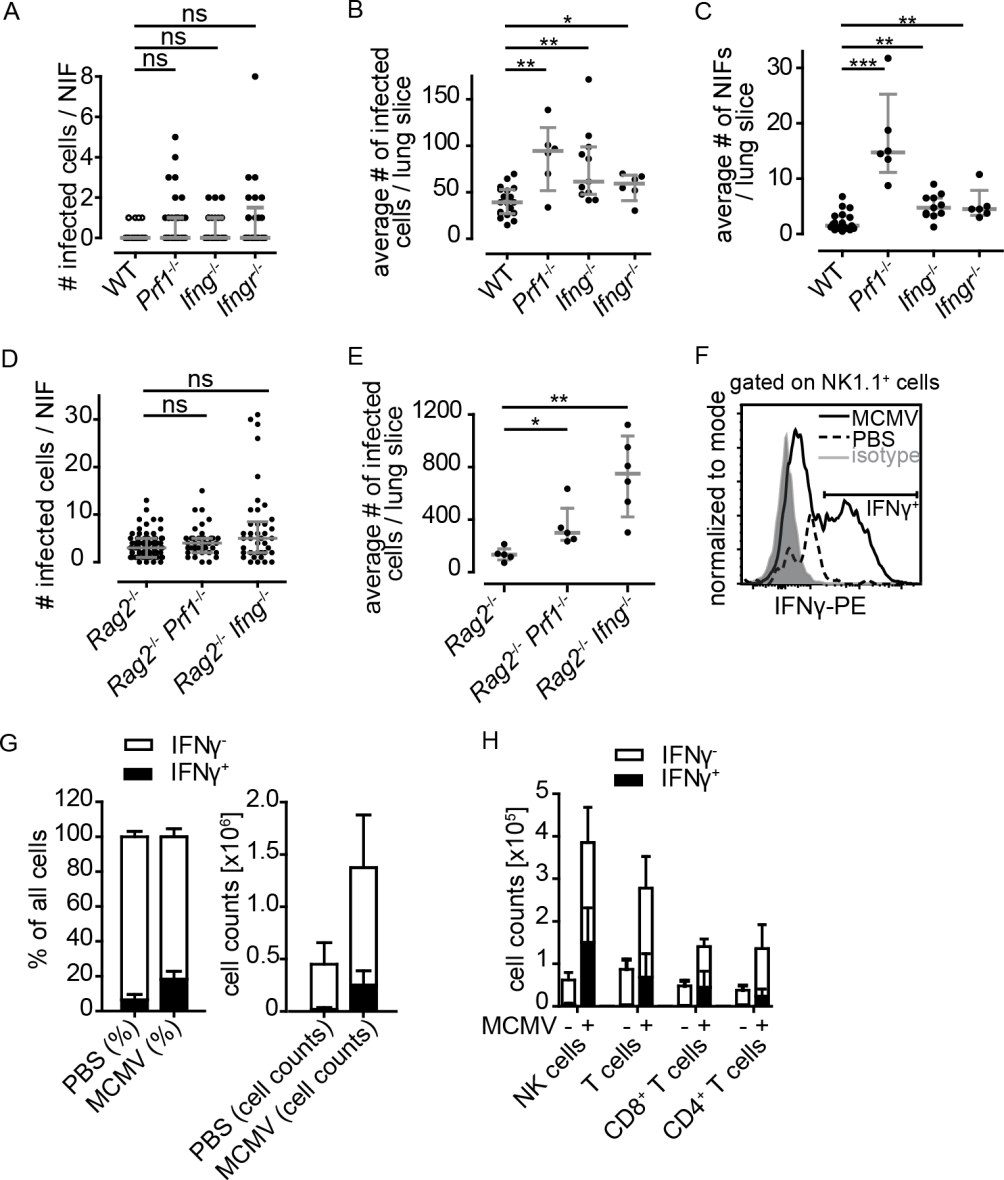
Perforin and IFNγ are potent factors controlling MCMV infection in the lung. **(A-C)** Mice deficient for either perforin, IFNγ or IFNγR1 or wild-type mice were infected i.n. with 10^6^ MCMV-3D. Quantification of the number of **(A)** infected cells per NIF, **(B)** infected cells per lung slice, or **(C)** NIFs per lung slice at 8 dpi. **(D+E)** Quantification of the number of infected cells per **(D)** NIF and **(E)** lung slice 8 dpi of genetically modified mice as indicated. Dots represent **(A+D)** NIFs, **(B, C, E)** means of 4 lung slices per animal. Mann-Whitney test performed with mean values of individual animals in comparison to **(A-C)** WT mice or **(D+E)** *Rag2*^-/-^ mice. **(A)** Open circles (WT) are identical to that shown in Fig 1C and 1D and Fig 3B, **(A-E)** Data pooled from 2–4 independent experiments; median + interquartile range, (grey). **(F-H)** Flow cytometric analysis of lung cells isolated from MCMV-infected or untreated wild-type mice 5 dpi after re-stimulation with PMA and ionomycin. **(F)** Representative histogram for IFNγ staining of NK1.1+ cells acquired. Gates were set based on isotype control (grey). **(G)** Percentages and absolute cell counts of all lung cells acquired positive (filled columns) or negative (open columns) for IFNγ of infected mice or PBS treated mice. **(H)** Absolute cell numbers of different cell populations positive (filled columns) or negative (open columns) for IFNγ in dependence on MCMV infection. **(G+H)** Mean + SD; data pooled from 4 animals per condition from 2 independent experiments.

To screen whether perforin and/or IFNγ might be essential for antiviral immunity in MCMV-infected lungs, we analyzed mice deficient for perforin (*Prf1*^-/-^), IFNγ(*Ifng*^-/-^), or IFNγ-receptor-1 (*Ifngr1*^-/-^). At 8 dpi, we quantified the number of infected cells per NIF but did not find any significant differences between WT and the three knock-out strains tested (Fig 5A). Additionally, we analyzed the number of intact infected cells per whole lung slice and found an increase of intact infected cells in *Prf1*^-/-^, *Ifng*^-/-^, and *Ifngr1*^-/-^ mice (Fig 5B) as well as an increase in the number of NIFs per lung section (Fig 5C). Compared to mice lacking T and B cells (Fig 3C), the differences of infected cell numbers observed here were less pronounced. Thus, these data indicate that antiviral effector mechanisms in NIFs of perforin- or IFNγ deficient mice may substitute for each other.

Next, we investigated the impact of IFNγ and perforin in the absence of T and B cells. To this end, we analyzed *Rag2*^-/-^*Prf1*^*-/-*^ and *Rag2*^-/-^*Ifng*^*-/-*^ mice 8 dpi. We found a slightly increased number of infected cells per NIF in *Rag2*^-/-^*Prf1*^-/-^ mice when compared to *Prf1*^*-/-*^ or *Rag2*^-/-^ mice (Fig 5A and 5D), while the number of infected cells per lung slice significantly increased in these mice (Fig 5B–5E). Furthermore, in *Rag2*^*-/-*^*Ifng*^*-/*-^ mice, an even higher number of intact infected cells was observed per lung slice, when compared to *Rag2*^*-/-*^ or *Ifng*^*-/-*^ single knock-out mice (Fig 5B and 5E). These findings support the idea that perforin as well as IFNγ can both play a role in controlling acute MCMV infection in the lungs. In absence of T cells, the secretion of these two molecules, most probably by NK cells, gains more importance in controlling the virus. However, it is likely that in *Rag2*^*-/-*^ and *Rag2*^-/-^*Il2rg*^-/-^ mice IFNγ is also produced by cells that do not express classical NK cell biomarkers [35].

To further characterize the cells producing IFNγ, we compared cytokine production of leukocytes isolated from MCMV- or mock-infected lungs following *in vitro* re-stimulation (Fig 5F). In total we observed more IFNγ-producing cells in infected animals than in control mice (Fig 5G). At 5 dpi, NK cells were found to be the dominant cell population to produce IFNγ followed by CD8 and CD4 T cells (Fig 5H). Interestingly, by histology less NK cells were found to be present in NIFs at 5 dpi, while flow cytometric analysis showed NK cells to be present in higher numbers in the lung (Fig 5H and S5 Fig). This indicates that most of the NK cells are present in the lung tissue but do not remain or proliferate in NIFs as efficiently as T cells. Taken together, NK and T cells were the major IFNγ-expressing cell types in the lungs during the peak of anti-MCMV immune response in an immunocompetent host leading to control of infection.

### IFNγ reduces viral cell-to-cell spread *in vitro*

**Fig 6:**
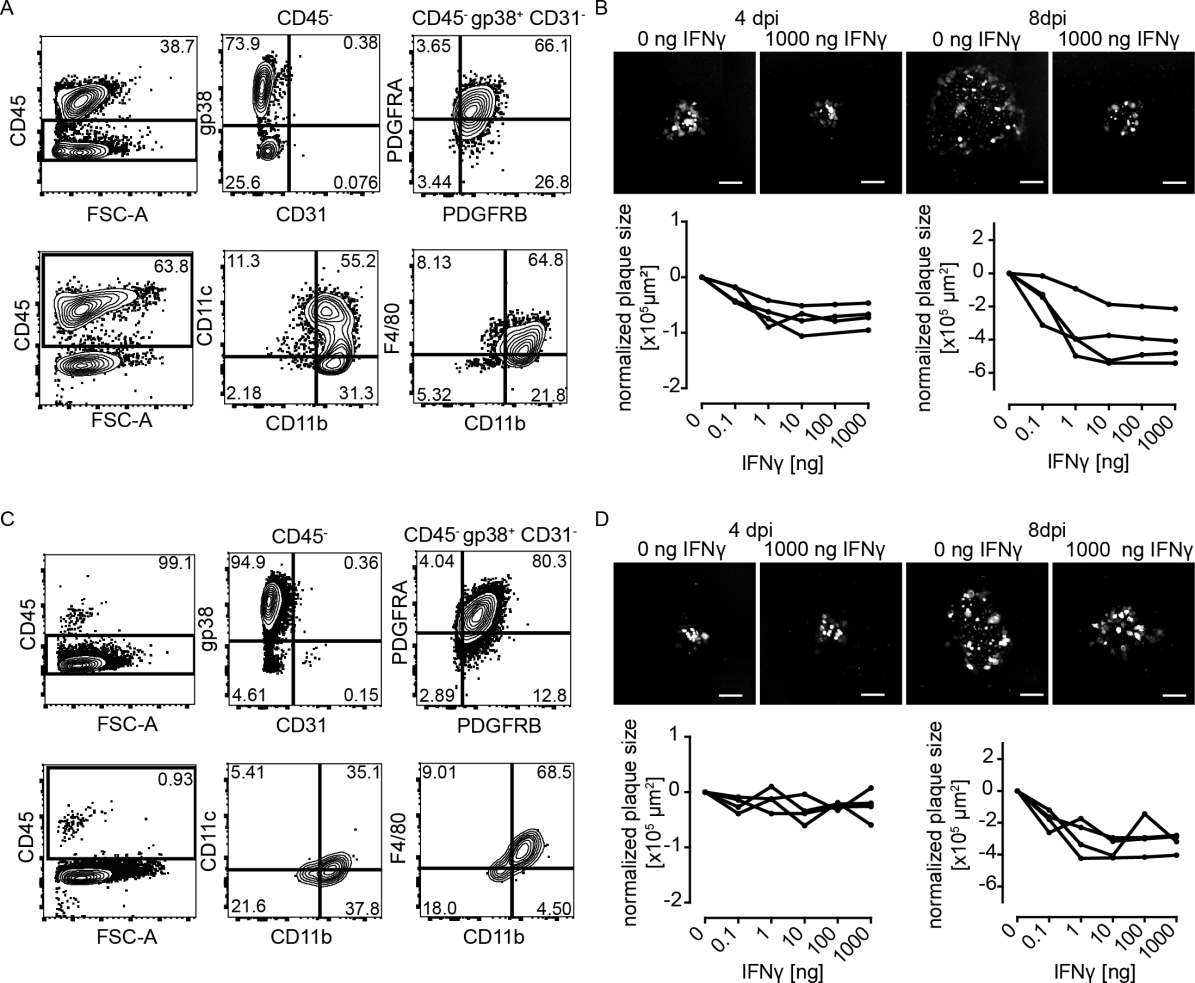
IFNγ reduces virus plaque growth on primary lung stromal cell cultures in the presence and absence of macrophages. Primary lung stromal cells were cultured in the **(A+B)** presence or **(C+D)** absence of lung-derived CD45^+^ cells. Cells were cultured for 13 days until reaching a confluent layer and subsequently infected with 20 PFU per well MCMV-3D. After infection cells were cultured in carboxymethylcellulose and the presence of different concentrations of mouse IFNγ. **(A+C)** Phenotyping of stroma cells cultured for 13 days. Characterization of the CD45 negative (upper row) and positive (lower row) population. **(B+D)** Measurement of plaque sizes. 4 and 8 dpi in the presence of different doses of IFNγ. Representative pictures of plaques in the absence or the presence of the highest dose of IFNγ used in these experiments (upper row) are shown; mCherry expressed by infected cells (white). Scale bar: 200 μm. Lower row: Mean of plaque sizes measured per well over different IFNγ doses (black curve, curve = single experiment; normalized on control wells without IFNγ of pooled experiments). See also S7 Fig.

To test how IFNγ could change the dynamics of MCMV spread in NIFs, we next modeled the situation in the lungs applying an *in vitro* plaque reduction assay using primary lung stromal cells. First, we purified adherent cells from the lungs and found approximately 40% of these cells to be gp38^+^PDGFRA^+^PDGFRB^+^CD31^-^ stromal cells (Fig 6A). The remaining cells were CD45^+^ hematopoietic cells, mainly of the myeloid lineage (CD11b^+^, partly CD11c^+^ and F4/80^+^; Fig 6A). Next, we infected this cell mixture *in vitro* with MCMV and followed the formation of plaques after addition of a single dose of recombinant IFNγ. At 4 and 8 dpi, we found that administration of IFNγ reduced plaque size in a dose-dependent manner (Fig 6B). Since about 60% of the cells in the culture were myeloid cells, the slower plaque growth might also be due to the activating effect of IFNγ on these myeloid cells. To investigate a direct effect of IFNγ on virus replication, we purified stromal cells by depletion of CD45^+^ cells. The resulting cell suspension lacked hematopoietic cells (Fig 6C). In this setup, we found IFNγ to have minor effects on plaque size at 4 dpi (Fig 6D). However, after 8 days, smaller plaques were observed in IFNγ-treated cell cultures in comparison to control cultures suggesting a direct interference of IFNγ with virus replication (Fig 6D). As control, we used stromal cells isolated from *Ifngr1*^-/-^ mice and found no effect for any of the IFNγ concentrations tested (S7 Fig). Together, these data indicate that IFNγ can slow down cell-to-cell spread in lung stromal cells. The difference observed in stromal cell cultures in the presence or absence of hematopoietic cells might be caused by indirect mechanisms mediated by IFNγ treated myeloid cells [36].

### T cells need IFNγ but not perforin to control acute MCMV infection in the lungs

**Fig 7:**
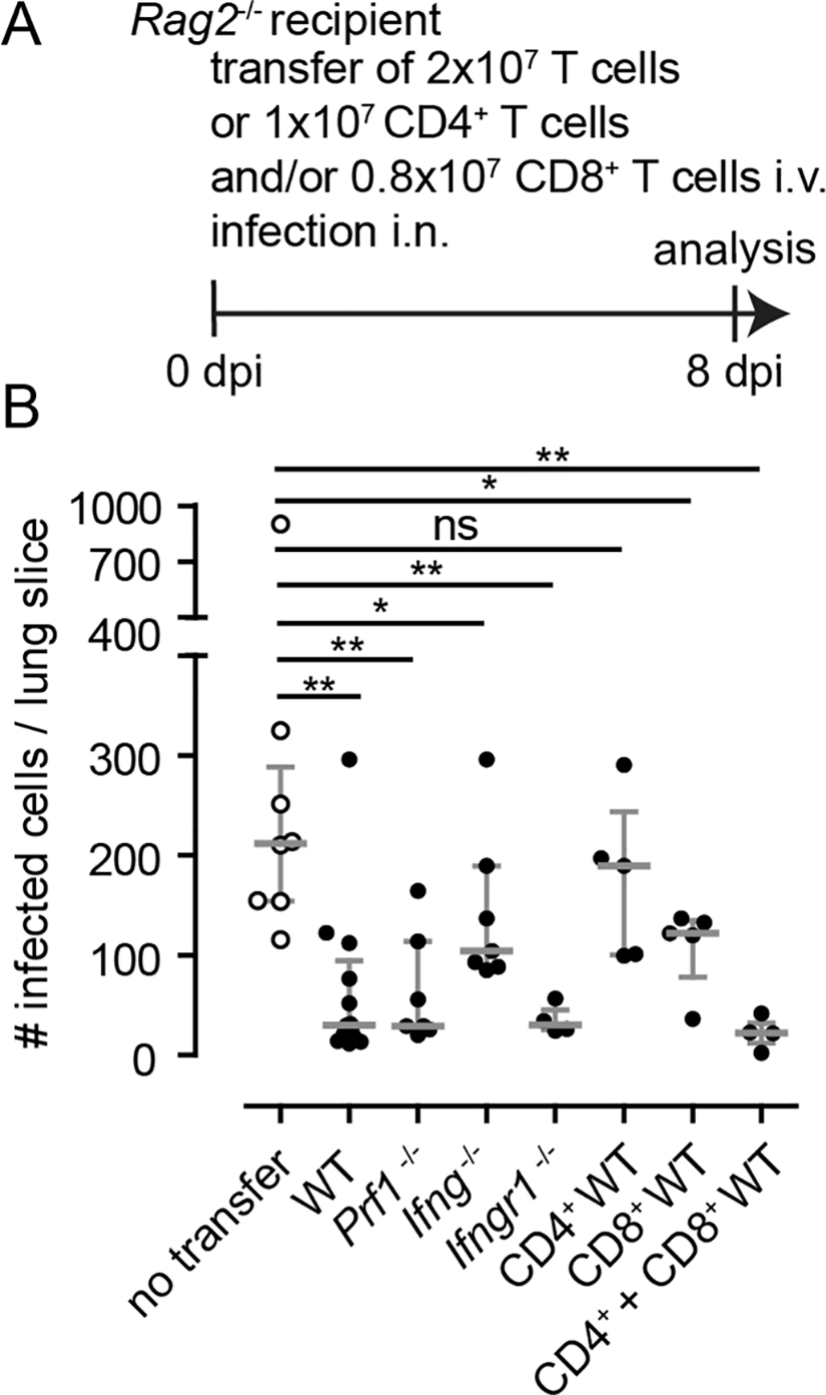
T cells need IFNγ to efficiently control infection in the lungs and CD8 T cells alone can only clear infection to some degree. **(A)** 2x10^7^ T cells, isolated from wild-type, *Ifng*^-/-^, *Ifngr1*^-/-^ or *Prf1*^-/-^ donors, or 1x10^7^ CD4 T cells and/or 0.8x10^7^ CD8 T cells of wild-type donors were adoptively transferred into *Rag2*^-/-^ recipients that were simultaneously infected with 10^6^ PFU MCMV-3D. Organs were analyzed at 8 dpi. **(B)** Quantification of the number of infected cells per lung slice. For comparison data from infected *Rag2*^-/-^ mice without T cell transfer are depicted in open circles (same data set as shown in Fig 3). Dots represent animal (mean of 4 lung slices per animal); median + interquartile range (grey); Mann-Whitney test compared to *Rag2*^-/-^ T cells without T cell transfer; Data from 2–4 independent experiments.

It is generally believed that IFNγ and perforin can inhibit local viral spread in the lungs. We also showed that NK and T cells were the major sources of IFNγ in MCMV-infected lungs. Therefore, we next asked whether T cells deficient for IFNγ or perforin could still control MCMV infection. Following adoptive transfer of naïve T cells isolated from wild-type, *Ifng*^-/-^, *Ifngr1*^-/-^, or *Pfr1*^-/-^ donor mice into *Rag2*^-/-^ recipients, we quantified the number of infected cells per lung (Fig 7A). At 8 dpi, the transfer of wild-type T cells reduced the number of infected cells per lung slice (Fig 7B) as observed before (Fig 4E). Importantly, following the transfer of 2x10^7^ IFNγ-deficient T cells, the control of MCMV infection was limited with many intact infected cells still detectable at 8 dpi (Fig 7B). In contrast, T cells deficient for the IFNγ-receptor showed a comparable antiviral effect to wild-type T cells, indicating that IFNγ signaling on T cells is not essential for T cells to suppress virus replication. Surprisingly, perforin-deficient T cells also reduced the number of infected cells (Fig 7B). Thus T cells need to secrete IFNγ to efficiently control MCMV infection in the lungs.

Finally, we asked whether helper or cytotoxic T cells are responsible for the T cell-mediated control of MCMV infection in the lungs. To address this question, we transferred CD4 and CD8 T cells isolated from naïve wild-type donors, separately or together, into MCMV-infected *Rag2*^-/-^ recipients (Fig 7A). We found that CD4 T cells in the absence of CD8 T cells were unable to suppress virus replication (Fig 7B). In contrast, CD8 T cells in absence of CD4 T cells had a detectable antiviral effect (Fig 7B). However, only the combination of CD4 and CD8 T cells lead to effective control of the infection indicating a cooperative effect of these two cell populations (Fig 7B). Taken together, these data suggest that i) for T cells secretion of IFNγ is essential to interfere with local MCMV replication in NIFs, ii) IFNγ-receptor signaling on T cells is not necessary to mediate their antiviral effect and iii) CD4 and CD8 T cells work together to fight virus infections in MCMV-infected lungs.

## Discussion

The immune response initiated by CMV infection can protect the host from severe disease. However, several immunosuppressive conditions can lead to recurrence of an unnoticed primary infection and during pregnancy primary infection or reactivation can lead to vertical transmission of the virus to the neonate [18]. Therefore, it is important to understand why the immune system can control, but not eradicate CMV infection in different organs. There are still many uncertainties regarding the antiviral factors needed to control CMV infection. In the mouse model, a classical read-out to assess the viral load of an organ is to determine viral titers via plaque assays or quantification of reporter virus-expressed luciferase activity within an organ of interest. Although these approaches can give a general overview of the viral load in individual organs, the localization of infected cells, the infected cell type, and the micro-anatomical context remain enigmatic.

In the present study, we combined a MCMV reporter mutant with various genetically modified mice to study the immune response to lung infection *in vivo*. The use of a reporter virus encoding the red fluorescent protein mCherry allows for simultaneous quantification of the infection as well as visualization of the immune cell-mediated antiviral response. Previously, we applied this method to assess the killing capacity of specific effector CD8 T cells [10] and to study differences between MCK2-proficient and -deficient virus mutants [27] as well as the immune responses in lungs of neonatal mice [26].

For the lungs, it has been reported that CMV infection causes pneumonia in immunocompromised humans [18] as well as in mice [29]. Focal infiltrations of immune cells following CMV infection have been observed in mice [29,37] and humans [28] and characterized as NIFs [26]. NIFs have been described as sites of infection but also as the site of antiviral immune response and we proposed recently that NIFs act as sites for T cell priming, at least in neonatal mice [26]. Thus, it is still unclear how NIFs are formed and how these structures contribute in detail to MCMV control in the lungs. Here, we showed that NIF formation is independent of the presence of lymphocytes and identified T cells as essential contributors to the antiviral effects observed within NIFs. Presumably, the recruitment of myeloid cells is triggered by the infected cells directly or by resident cells in close proximity to infected cells. In parallel to this first wave of cellular influx lymphocytes locate in forming NIFs to mediate their antiviral effect directly at the site of infection. Once the infection is kept under control within NIFs the pro-inflammatory signals vanish and the structures dissolve to allow tissue healing. In the liver, CCL3 was reported to be essential for the infiltration of immune cells after MCMV infection [38], while CCL5 was proposed as a possible recruitment factor for T cells into the lung interstitium [39,40]. However, the factors inducing and sustaining NIFs in the lungs and chemokines involved in recruiting immune cells to these structures need further investigation.

Primary MCMV infection of the adult lungs can be controlled within approximately one week in immunocompetent mice [26]. In contrast to wild-type mice, we find that various strains of immunocompromised mice are impaired in reducing the number of infected cells in the lungs. NIFs with intact infected cells are still detectable 8 dpi in immunocompromised but not in wild-type mice. Although we used MCMV reporter viruses deficient for m157 that encodes a ligand for the NK activating receptor Ly49H [22], our results indicate that NK cells contribute to virus control in NIFs even in the absence of this strongly activating ligand especially in a T cell deficient host. Therefore, the NK cell-mediated contribution to virus control must be induced by other mechanisms that have not been addressed here. In fact, we found NK cells to be the major population of lymphocytes able to secrete IFNγ at 5 dpi. Notably, an IFNγ-producing cell population lacking T and NK cell surface markers has been identified in *Rag1*^-/-^ and *Rag2*^*-/-*^*Il2rg*^*-/-*^ mice [35]. Furthermore, the secretion of perforin might be crucial for the NK cell mediated control, since we found increased virus loads in perforin-deficient mice while this effect was apparently not T cell-mediated.

Using various experimental approaches, we show in lungs that T cells have a major effect on NIF dynamics and virus control in NIFs. Effector CD8 T cells are known to essentially contribute to the control of MCMV infection in mice [41–44] and humans [4] and adoptive transfers of specific T cells are applied as clinical therapy for several years now [7]. However, so far the efficacy of T cells controlling the primary infection in the lungs of immunocompetent mice has not been addressed in detail. We found that T cells migrate into NIFs as early as these structures can be detected (3 dpi); with an increasing number of T cells being present until 8 dpi. The observed increase of T cell counts in NIFs can be either due to enhanced recruitment to the site of infection or to local proliferation following priming in NIFs—as shown before in neonatal mice [26]–or both. Earlier studies revealed that the adoptive transfer of 10^5^−10^7^ T cells (either activated lymphocytes from lymphoid organs, or pulmonary T cells, or pulmonary CD8 T cells from previously infected donors) were sufficient to reduce the viral titer or the amount of infected cells in several organs of infected immunocompromised recipients [42–44]. In contrast, our data indicate that only the adoptive transfer of higher numbers of naïve T cells (10^7^) led to T cell densities in NIFs that contributed to sufficient local virus control to a similar degree as observed in WT mice 8 dpi. This high number of polyclonal naïve T cells is essential to provide sufficient numbers of precursors for MCMV-specific T cells that can be expanded and subsequently control the infection.

The role of CD4 T cells in controlling cytomegalovirus infection has been discussed controversially. Several adoptive transfer experiments showed that CD4 T cells on their own cannot control MCMV infection [42,45,46]. Nevertheless, CD4 T cells have been shown to be necessary to control MCMV in the salivary glands [16,17] and other organs [47] in mice and clinical case reports revealed that the presence of CD4 T cells might be crucial for efficient viral control in patients [7,48–53].

Applying adoptive transfers into *Rag2*^-/-^ mice, we showed that CD4 T cells support CD8 T cells in controlling virus infection in the lungs. This effect might be mediated by a type of T helper-1 function supporting CD8 T cell differentiation as suggested elsewhere [54] but it seems also likely that CD4 T cells possess cytotoxic effector functions directly contributing to the control of infection [7,55]. Intracellular staining for IFNγ revealed that around 20% of the CD4 T cells secrete IFNγ indicating that a considerable proportion of the CD4 T cells show a T helper-1 phenotype.

Furthermore, we showed that the secretion of IFNγ by T cells was essential to reduce the number of infected cells while the absence of perforin did not affect the T cell mediated control. IFNγ has been reported to have multiple effects on different cell types [56–58]. For example, IFNγ drives differentiation of monocytes into macrophages that mediate anti-microbial effects [59]. Likewise the function of the myeloid cells abundantly present within NIFs might also be affected by the locally secreted IFNγ. In an *in vitro* assay we found that IFNγ affected plaque size of lung stromal cells after MCMV infection, indicating that IFNγ stimulates intrinsic cell mechanisms to interfere with virus replication [60,61]. Interestingly, not only pretreatment but also treatment of cells with IFNγ after an initial MCMV infection can result in an inhibiting effect on the viral spread [36]. It is known that IFNγ can counteract to some degree MCMV-mediated down-modulation of MHC I that in turn might directly facilitate CD8 T cell-mediated killing [62]. Previously, we reported that the presentation of viral antigen via MHC I by MCMV-infected target cells and the following direct recognition of the infected cell by antigen-specific effector CD8 T cells is essential for the cytotoxic killing [10]. In addition to IFNγ and perforin other factors, such as TNFα and Fas/FasL, play an important role in the T cell effector function and must be addressed in similar models in the future.

Based on the data presented in this manuscript we envisage the following model how T cells contribute to the local control of MCMV in lung NIFs: T cells are primed in lymphoid organs and within NIFs to secrete IFNγ directly at the site of infection. This secretion of IFNγ not only slows down the viral cell-to-cell spread but might also interfere with MHC I molecule downregulation on the surface of infected cells allowing for better recognition of viral antigen by MCMV-specific CD8 T cells. Furthermore, the IFNγ secretion by NK, as well as both CD4 and CD8 T cells, can stimulate macrophages to efficiently phagocytose infected cell remnants and clear the tissue from infectious virus particles. In parallel, NK cells contribute to MCMV control in a perforin-dependent manner. Thus, a combination of virus-specific CD8 and CD4 T cells, with the addition of NK cells, might be an optimal recipe for anti-CMV adoptive immunotherapy.

## Materials and methods

### Ethics statement

All animal experiments were performed according to the recommendations and guidelines of the Federation of European Laboratory Animal Science Associations and Society of Laboratory Animals and approved by the institutional review board and the Niedersächsische Landesamt für Verbraucherschutz und Lebensmittelsicherheit (33.12-42502-04-10/0225, 33.12-42502-04-12/0921, 33.12-42502-04-13/1255, and 13.12-42502-04-17/2737). All experiments were performed according to the Tierschutzgesetz and the Tierschutz-Versuchstier-Verordnung. Anesthesia of mice was performed with Ketamin and Xylazin injection. Mice were sacrificed following CO_2_ anesthesia by cervical dislocation.

### Animals

Mice were bred at the central animal facility of Hannover Medical School under specific pathogen free conditions. The different mouse strains (*Ighm*^*tm1Cgn*^ [63], *Cd3e*^*tm1Mal*^ [64], *Rag2*^*tm1*^ [65], *Rag2*^*tm1*^*Il2rg*^*tm1*^[66,67], *Ifng*^*tm1Ts*^ [68], *Ifngr1*^*tm1Agt*^ [69], *Prf1*^*tm1Sdz*^ [70], *Ncr1*^gfp/wt^ [71]) were maintained on a C57BL/6 (B6) background. *Rag2*^-/-^*Prf1*^-/-^ and *Rag2*^-/-^*Ifng*^-/-^ mice were generated by intercrossing *Rag2*^-/-^ and *Prf1*^-/-^ or *Rag2*^-/-^ and *Ifng*^-/-^ mice, respectively. 6–14 weeks old mice were used for the experiments. All mouse experiments were performed in accordance with local animal welfare regulations.

### Virus, infection and total body gamma-irradiation

The MCMV strain named MCMV-3D has been described previously [20]. Virus stocks were produced and titrated on mouse embryonic fibroblasts. MCMV-3D encodes Gaussia luciferase and mCherry and carries an additional sequence within the m164 ORF encoding the SIINFEKL peptide. It lacks the m157 ORF that encodes a ligand for the activating receptor Ly49H present on a subset of NK cells in C57BL/6 mice and expresses a truncated version of the viral MCK2 protein [27,30]. In some experiments, the reporter virus MCMV-3DR with a repaired *m129* ORF resulting in an intact MCK2 protein was used for intranasal infection [27]. All animals were infected intranasally with 10^6^ PFU MCMV-3D or MCMV-3DR. In some experiments C57BL/6 mice were irradiated with a total dose of 6 Gray in one irradiation procedure the day before intranasal infection.

### Adoptive cell transfer and antibody depletion

In experiments *Rag2*^-/-^ mice were used as recipients of adoptive T cell transfers. T cells were isolated from lymph nodes and spleens of untreated wild-type B6, *Ifng*^-/-^, *Ifngr1*^-/-^, or *Prf1*^-/-^ mice using MACS pan T cell, CD4 T cell, or CD8 T cell negative selection kits (Miltenyi Biotech) and adoptively transferred intravenously in parallel to MCMV infection. For *in vivo* depletion of NK cells in B6 and *Rag2*^-/-^ mice 300 μg anti-NK1.1 (antibody clone PK136) was applied intraperitoneally four hours before and every second day after MCMV infection.

### Virus titers and luciferase assay

Organs were perfused *in situ* with cold PBS, explanted and stored in 500μL DMEM containing 10% FCS and 1% P/S. Next, the organs were mechanically disrupted by metal beads and shaking at 25/s over 4 min (TissueLyser II, Quiagen). Then, 350 μL of the homogenate were separated and stored at -80°C for viral plaque titration, while the rest was centrifuged at maximum speed for 15 min. 20 μL of the substrate (1 μg Coelenterazine / ml in PBS) were added freshly to 180μL of a 1:10 dilution of the organ homogenate and measured for 10s directly with Lumat LB 9507 (Berthold Technologies) in duplicates. The viral plaque titration was performed on MEF monolayers in triplicates.

### Histology

Mice were sacrificed and lungs were perfused with cold PBS via the right heart ventricle and then filled *in situ* with PBS solution containing 2% paraformaldehyde and 30% sucrose. Fixed lungs were embedded in OCT compound (TissueTek, Sakura) before freezing at -20°C.

7 μm thick cryosections were blocked with rat serum in TBST and stained with antibodies and nuclei were stained with DAPI. The following antibodies (clones) were used after additional blocking of Fc receptors with rat anti-CD16 and anti-CD32 (2.4G2): CD11b-bio (MAC1), CD3-Cy5 (CD317A2), CD11c-APC (N418), CD45.2-FITC (104), CD45-APC (30-F11), and CD8a-PE (53–6.7). For staining with anti-mouse NK1.1-APC (PK136) and GFP-binding protein (GFP-boost, Chromotek), rehydration was performed in 0.5% Triton-X following blocking with mouse serum and Fc receptor blocking, while slides were stained in 1%Triton-X. Images were taken with an AxioCam MRm camera (Carl Zeiss) attached to an Axiovert 200M fluorescence microscope (Carl Zeiss) with PlanApochromat objectives 10x/0,45 and 20x/0,75 (magnification/numerical aperture) and with AxioScan.Z1 (Carl Zeiss) and processed with AxioVision 4.8.2 software and with ZEN blue 2.3 software.

### Quantification of cells from lung histology

Lung sections were analyzed at defined anatomical positions (through the center; slices included right and left lobes as well as main bronchi). Sections were stained with DAPI. For each animal, 4 lung sections were analyzed by an observer blinded for mouse identity. Manual counting of virus-infected cells and NIFs was performed directly at the microscope. Intact cells were distinguished from remnants of infected cells based on their mCherry expression, morphology, and DAPI staining while NIFs were quantified based on localized accumulation of nuclei as well as presence of mCherry signals. The number of independent experiments performed is indicated in each figure. In general, we only show data from at least 4 mice and at least 2 independent experiments. For the analysis of infected cells per NIFs, microscopy pictures were taken with the 20x/0.75 objective. T cells were quantified based on CD3 staining, CD8 T cells were quantified based on CD8a staining, and NK cells were quantified based on GFP expression in Ncr1^gfp/wt^ mice and additional staining with a GFP-binding protein in the area of infiltration that was semi-automatically measured based on CD45 staining using ImageJ. Between 10 and 15 NIFs were analyzed per animal.

### Flow cytometry and intracellular staining

Leukocytes from lungs were isolated as described before [26]. Briefly, right heart ventricle was perfused with PBS until blood cells were removed from the lungs. Fragmented tissue was digested with Collagenase D (Roche, 0.5 mg/ml) and DNAse I (Roche, 0.025 mg/ml) for 45 min at 37°C, meshed through 40 mm Falcon Cell Strainer and leukocytes isolated with Lympholyte-M (Cedarlane) gradient centrifugation technique. For tetramer staining, lung leukocytes were stained with a mix of APC-tetramers specific for MHCI complexes presenting M45, M38, or m139 for 15 min at 4°C, following a surface staining for 15 min on ice. Tetramers were provided by Ramon Arens (LUMC, Netherlands). For intracellular staining, lung leukocytes were cultured *in vitro* in 200 μL RPMI/FCS/HEPES. For re-stimulation 50 ng/mL PMA and 500 ng/mL Ionomycin were added and cells were incubated for 2 hours at 37°C with 5% CO_2_. To allow detection of intracellular proteins 10 μg/mL Brefeldin A were added and incubated for another 2 hours at 37°C and 5% CO_2_. Fc-receptors were blocked in PBS with 3% FCS (PBS/FCS), 5% serum, and rat antibodies to mouse CD16 and CD32 (2.4G2). Surface staining was performed for 15 min on ice following the fixation and intracellular staining procedure with the Intracellular Fixation & Permeabilization Buffer Set (eBiosciences) according to the manufacturer protocol. Cells were re-suspended in PBS/FCS for flow cytometry analysis using a LSRII (BD Biosciences). Flow cytometry data was analyzed using FlowJo 7.5 and 10. Gates for IFNγ^+^ cells were set based on the border of 99% of the isotype control. Cells from *in vitro* culture experiments were trypsinized at room temperature (for 3 min using PBS containing 0.5 mg/mL Trypsin and 3 mM EDTA), blocked for Fc-receptors and stained with antibodies before FACS analysis. Following antibodies (clones) were used for flow cytometry staining: CD8a-PE (53–6.7), CD8-APC (RmCD8-2), Thy1-Alexa488 (RMT1), CD103-FITC (M290), CD31-FITC (MEC13.3), Ly6G-PE (1A8), CD11b-eF450 (M1/70), CD45.2-APC (104), EpCAM-eF450 (G8.8), F4/80-APC-eF780 (BM8), gp38-bio (eBio8.1.1), Ly6C-PerCP-Cy5.5 (HK1.4), CD11c-PeCy7 (N418), CD19-BV510 (6D5), CD4-PerCP (RM4-5), CD45.2-PerCP-Cy5.5 (104), IFNγ-PE (XMG1.2), NK1.1-PeCy7 (PK136), Pdgfra-PE (APA5), and Pdgfrb-APC (APB5).

### *In vitro* plaque reduction assay

Animals were sacrificed and the right heart ventricle was perfused with cold PBS to remove blood cells from the lungs. Lungs were explanted, chopped into pieces and digested in RPMI with 0.125 mg DNAse I and 0.269 mg collagenase D at 37°C for 2h. Cell suspensions were treated with erythrocyte lysis buffer and stromal cells either purified by depletion of CD45^+^ cells with the use of a MACS CD45 positive selection kit (Miltenyi) or cultured as a mixed population. Cells were cultured in RPMI with FCS, P/S and L-Glutamine, and medium was exchanged every day to remove dead and non-adherent cells. After 6 days of culture, cells were trypsinized, seeded on 48 well plates and cultured for additional 7 days to reach a confluent layer. Cells were then infected with 20 PFU/well for 30 mins. Afterwards, virus solution was discarded and cells were covered with carboxy-methyl-cellulose in addition to different concentrations of mouse IFNγ protein (0, 0.1, 1, 10, 100 and 1000 ng per well). After 4 and 8 days, mosaic images were taken with Zeiss Axiovert M microscope, and plaque sizes were measured with ImageJ.

### Statistical analysis

Statistical analysis was performed using GraphPad Prism 4. A non-parametric two-tailed t-test (Mann-Whitney test) was applied to compare data from experimental groups to that of wild-type mice (Fig 2, Fig 3, Fig 5A–5C, S1 Fig, S2 Fig, S3 Fig, and S4 Fig) or of *Rag2*^-/-^ mice (Figs 4D, 4E, 5D, 5E and 7). In Fig 4F, the Wilcoxon signed-rank test was applied comparing experimental groups to a fixed value of 0, since *Rag2*^*-/-*^ do not harbor any T cells. Significant differences are marked as follows: P>0.05 (ns), P<0.05 (*), P<0.01 (**), P<0.001 (***).

## Supporting information

S1 Fig Viral titers and luciferase activities increase in salivary glands after i.n. infection and are restricted by hematopoietic cells. Animals were either irradiated with 6 Gy or not (control) and infected one day post irradiation with 10^6^ PFU MCMV-3D i.n. At 5 and 8 dpi salivary glands were homogenized and viral titers **(A)** or luciferase activities **(B)** were determined. Dots represent means of **(A)** triplicates or **(B)** duplicates per organ. **(A+B)** Median plus interquartile range are shown in grey; Mann-Whitney test. **(C)** Correlation of viral titers and luciferase activity in salivary glands. **r:** Spearman coefficient. **(A-C)** DL: detection limit; data from 2 independent experiments per time point are shown. PFU: plaque forming units; RLU: relative light units.
(TIF)

S2 Fig NIFs do not contract in T cell-deficient mice 8 dpi. Wild type mice and various mouse strains deficient for different immune cell populations were infected i.n. with 10^6^ PFU MCMV-3D. Quantification of NIF size at **(A)** 5 and **(B)** 8 dpi. Dots represent NIFs in 1–2 independent experiments; median + interquartile range (red); Kruskal-Wallis test with Dunn’s Multiple Comparison to WT-mice.
(TIF)

S3 Fig NK cells do not play a crucial role in containing MCMV infection in the presence of T cells. Animals were depleted or not for NK cells before i.n. infection with 10^6^ PFU MCMV-3D. PFA-fixed lung cryosections were prepared from mice and analyzed at 5 dpi. Infected cells were quantified per **(A)** NIFs and **(B)** lung section. Dots represent **(A)** NIFs or **(B)** means of 4 lung slices analyzed per animal. Median plus interquartile range shown in red; Mann-Whitney test performed with mean values of individual animals.
(TIF)

S4 Fig Control of MCK2-proficient MCMV in the lungs also relies on T cells. WT or *Rag2*^*-/-*^ mice were infected i.n. with 10^6^ MCMV-3DR (MCK2 proficient; red) or MCMV-3D (MCK2 deficient; black) and lungs were analyzed at 8 dpi. Infected cells were quantified per **(A)** NIF and **(B)** lung section. Dots represent **(A)** NIFs and **(B)** mean of 4 lung sections analyzed per animal; median + interquartile range (grey); Mann-Whitney test performed with mean values of individual animals.; data shown from 2 independent experiments.
(TIF)

S5 Fig Presence of NK and CD8 T cells within NIFs. Ncr1^gfp/wt^ mice were i.n. infected with 10^6^ PFU MCMV-3D. **(A)** Histology of representative NIFs GFP, blue; anti-CD8a, orange; anti-CD45, green; infected cells, red; scale bar 50 μm. **(B+C)** Quantification of the number of **(B)** NK cells and **(C)** CD8 T cells per NIF at 3 and 5 dpi. Dots represent NIFs; median + interquartile range; Mann-Whitney test. **(D+E)** Quantification of number of **(D)** NK cells or **(E)** T cells per NIF area; Dots represent NIFs.
(TIF)

S6 Fig MCMV-specific CD8 T cells are present in infected lungs. Animals were infected with 10^6^ PFU MCMV-3D i.n. and lungs were analyzed at 5 and 8 dpi. For FACS analysis cells were stained with a mixture of three different tetramers (M45, m139, and M38). **(A)** Number of tetramer^+^ cells per lung and **(B)** frequency of tetramer^+^ cells of CD8^+^ T cells. Data points represent animals; median + interquartile range (grey); data from 2 experiments shown.
(TIF)

S7 Fig IFNγ treatment does not affect plaque size of infected stromal cells of *Ifngr1*^-/-^ mice. Primary lung stromal cells of *Ifngr1*^-/-^ mice were cultured in the **(A+B)** presence or **(C+D)** absence of lung-resident CD45^+^ hematopoietic cells. Confluent cells were infected with 20 PFU MCMV-3D per well. After infection, carboxy-methyl-cellulose and various concentration of recombinant mouse IFNγ was added. Quantification of plaque sizes **(A+C)** 4 dpi and **(B+D)** 8 dpi after treatment with IFNγ as indicated. Mean of plaque sizes per well in dependence on the amount of IFNγ added (curves depict single experiments normalized to wells not treated with IFNγ). Data from 3–4 independent experiments are shown.
(TIF)
