# Supplementary figures and images for: Control of primary mouse cytomegalovirus infection in lung nodular inflammatory foci by cooperation of interferon-gamma expressing CD4 and CD8 T cells
Source: PLoS Pathog. 2018 Aug 28;14(8):e1007252. doi: 10.1371/journal.ppat.1007252 (PMC6112668; doi:10.1371/journal.ppat.1007252)

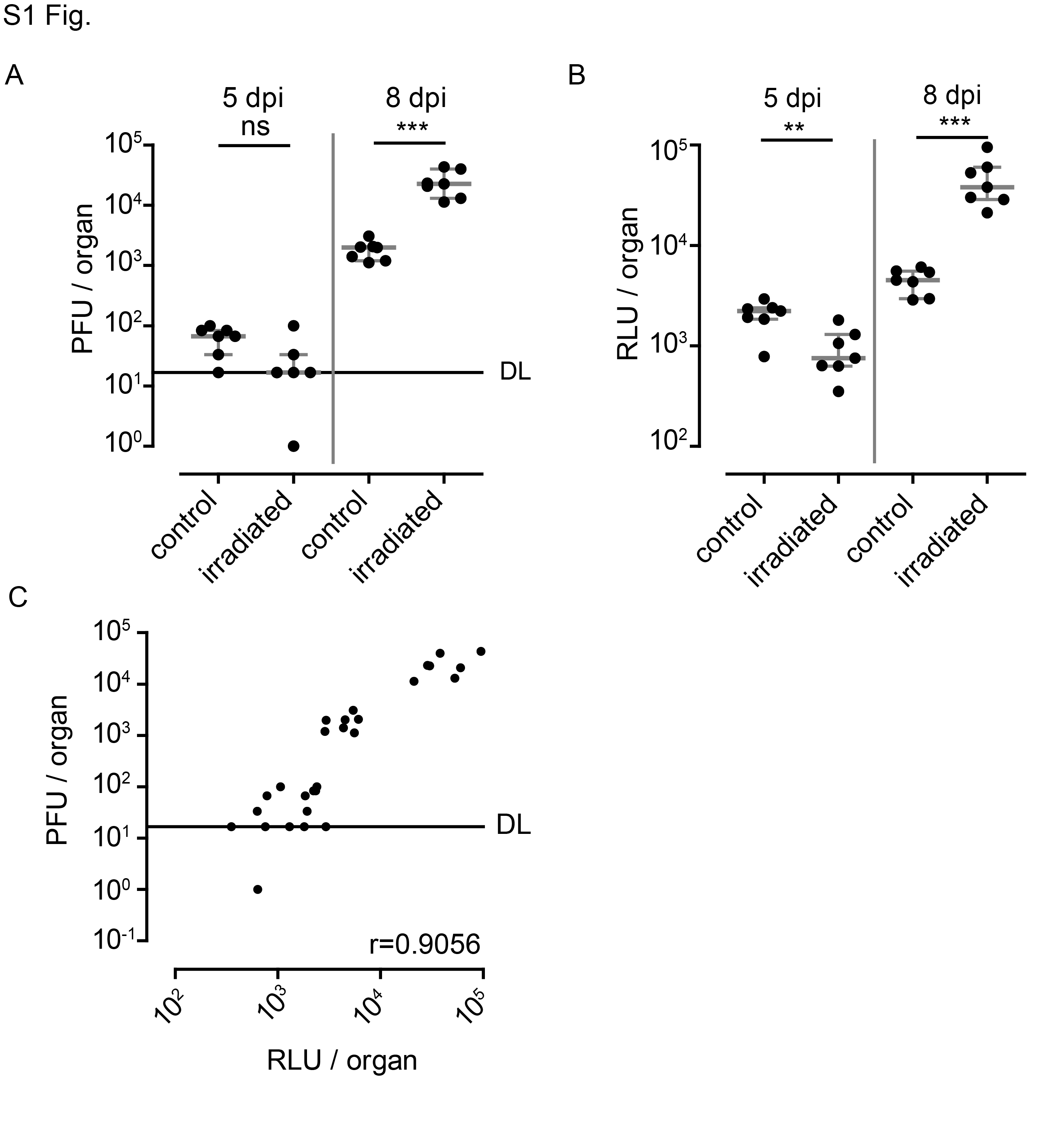

Supplement: S1 Fig — Animals were either irradiated with 6 Gy or not (control) and infected one day post irradiation with 106 PFU MCMV-3D i.n. At 5 and 8 dpi salivary glands were homogenized and viral titers (A) or luciferase activities (B) were determined. Dots represent means of (A) triplicates or (B) duplicates per organ. (A+B) Median plus interquartile range are shown in grey; Mann-Whitney test. (C) Correlation of viral titers and luciferase activity in salivary glands. r: Spearman coefficient. (A-C) DL: detection limit; data from 2 independent experiments per time point are shown. PFU: plaque forming units; RLU: relative light units. (TIF) [file ppat.1007252.s001.tif]

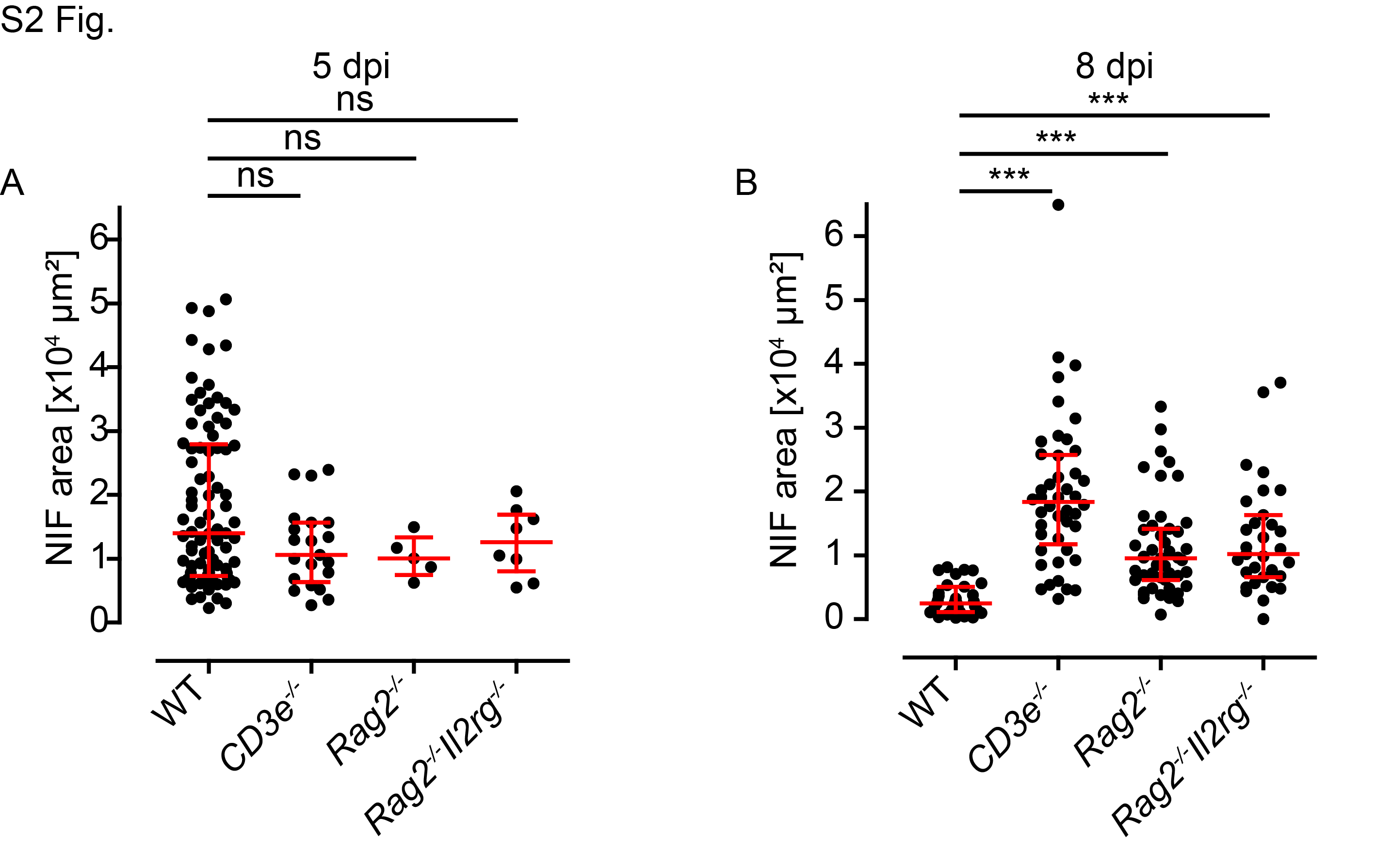

Supplement: S2 Fig — Wild type mice and various mouse strains deficient for different immune cell populations were infected i.n. with 106 PFU MCMV-3D. Quantification of NIF size at (A) 5 and (B) 8 dpi. Dots represent NIFs in 1–2 independent experiments; median + interquartile range (red); Kruskal-Wallis test with Dunn’s Multiple Comparison to WT-mice. (TIF) [file ppat.1007252.s002.tif]

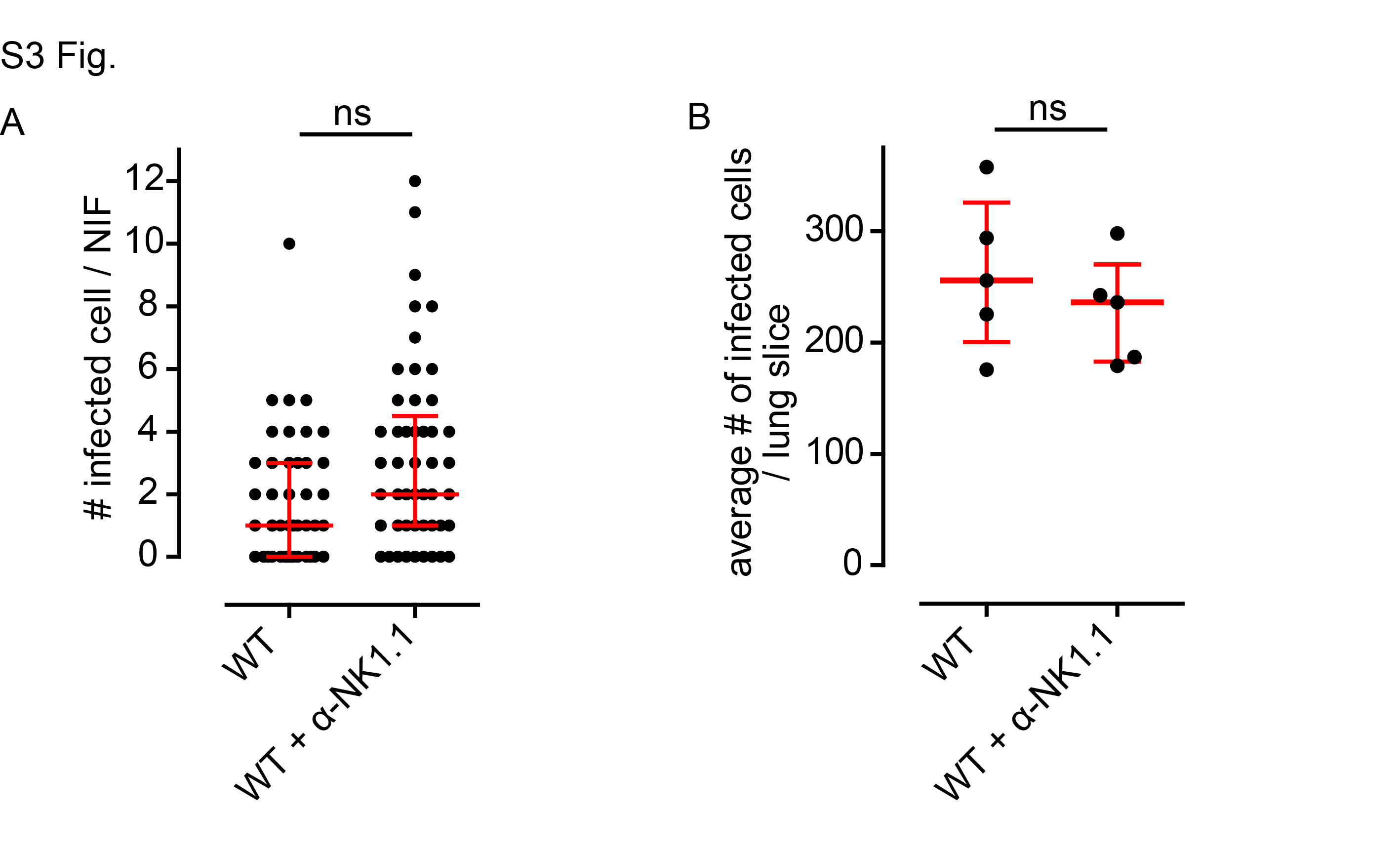

Supplement: S3 Fig — Animals were depleted or not for NK cells before i.n. infection with 106 PFU MCMV-3D. PFA-fixed lung cryosections were prepared from mice and analyzed at 5 dpi. Infected cells were quantified per (A) NIFs and (B) lung section. Dots represent (A) NIFs or (B) means of 4 lung slices analyzed per animal. Median plus interquartile range shown in red; Mann-Whitney test performed with mean values of individual animals. (TIF) [file ppat.1007252.s003.tif]

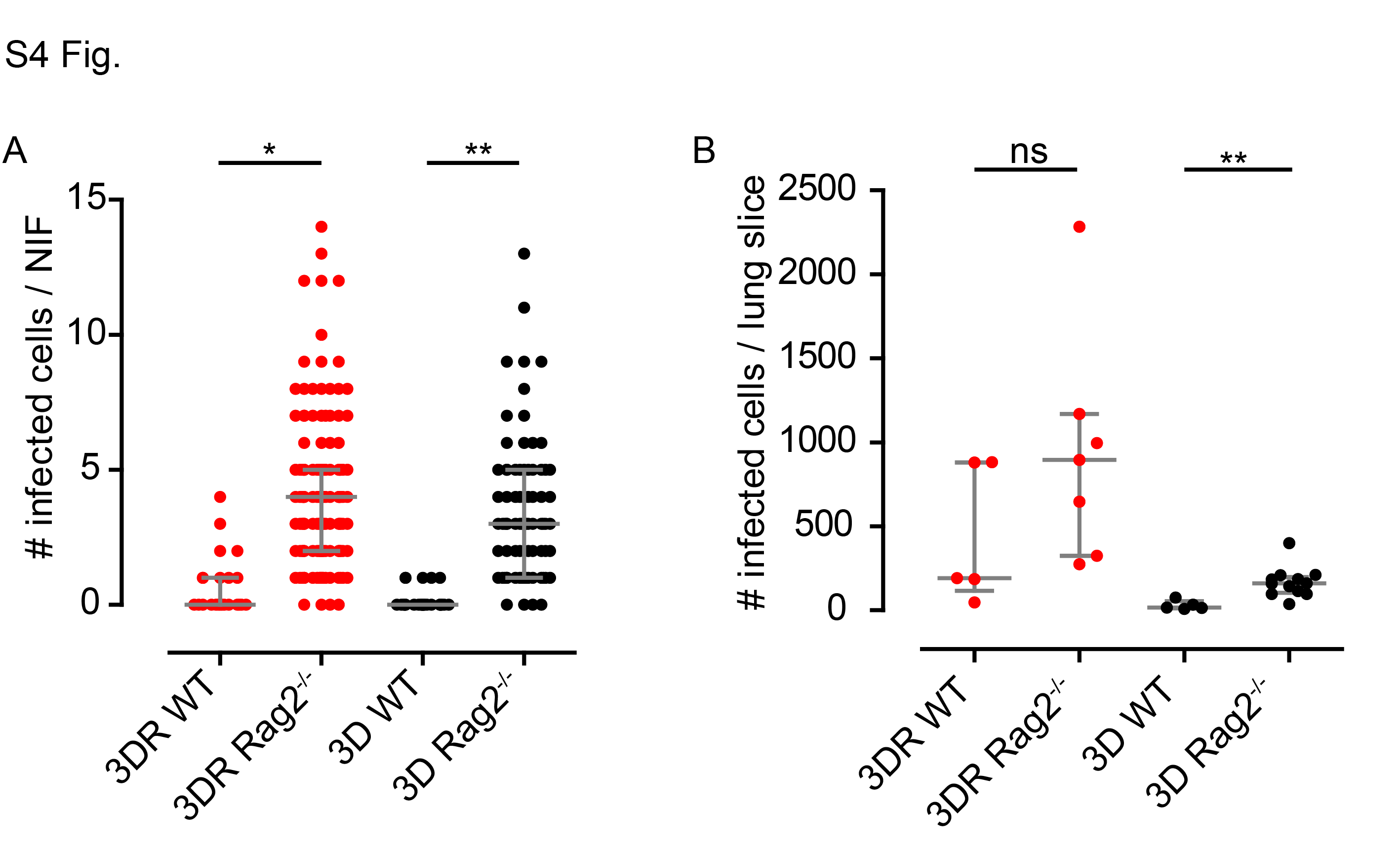

Supplement: S4 Fig — WT or Rag2-/- mice were infected i.n. with 106 MCMV-3DR (MCK2 proficient; red) or MCMV-3D (MCK2 deficient; black) and lungs were analyzed at 8 dpi. Infected cells were quantified per (A) NIF and (B) lung section. Dots represent (A) NIFs and (B) mean of 4 lung sections analyzed per animal; median + interquartile range (grey); Mann-Whitney test performed with mean values of individual animals.; data shown from 2 independent experiments. (TIF) [file ppat.1007252.s004.tif]

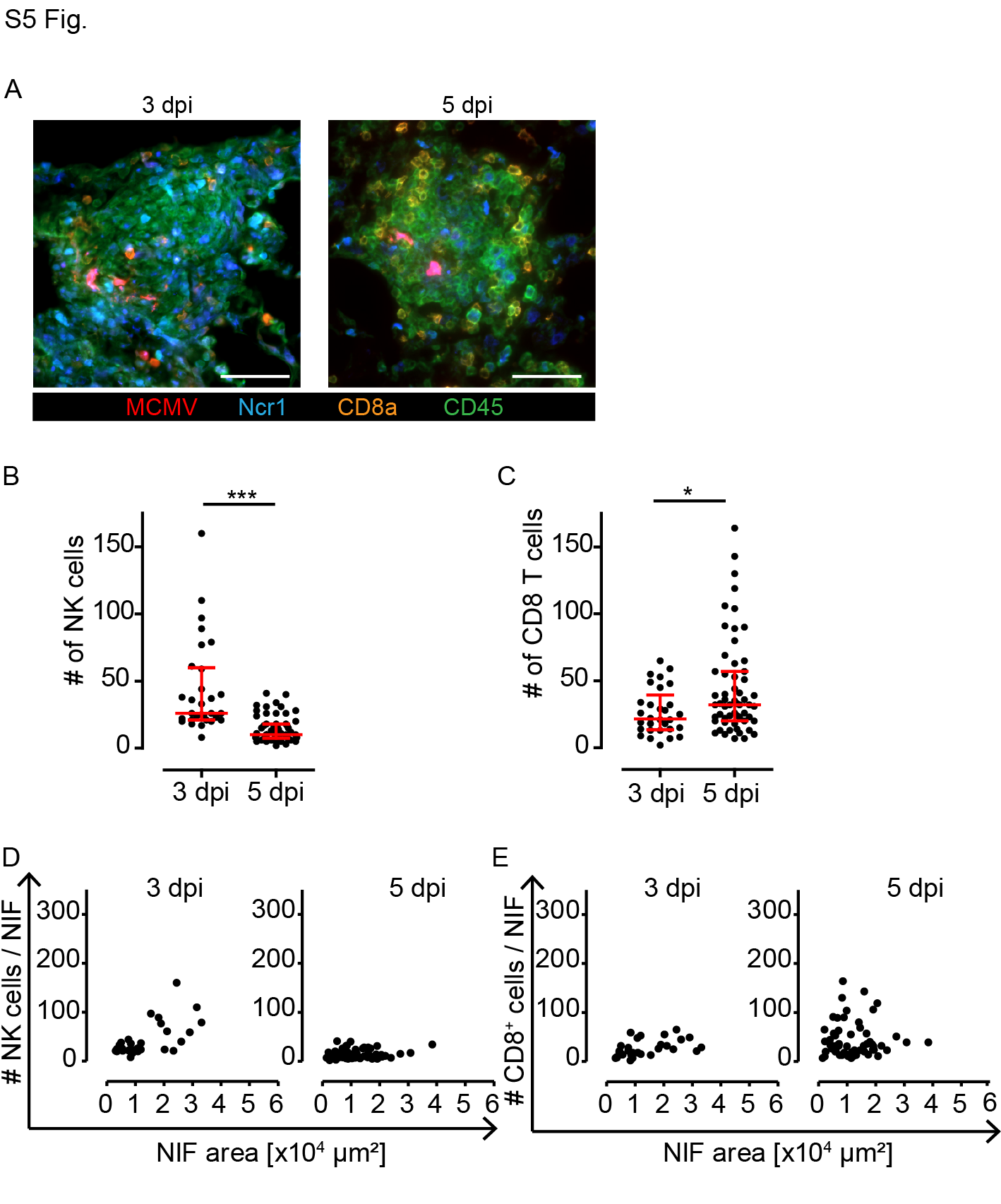

Supplement: S5 Fig — Ncr1gfp/wt mice were i.n. infected with 106 PFU MCMV-3D. (A) Histology of representative NIFs GFP, blue; anti-CD8a, orange; anti-CD45, green; infected cells, red; scale bar 50 μm. (B+C) Quantification of the number of (B) NK cells and (C) CD8 T cells per NIF at 3 and 5 dpi. Dots represent NIFs; median + interquartile range; Mann-Whitney test. (D+E) Quantification of number of (D) NK cells or (E) T cells per NIF area; Dots represent NIFs. (TIF) [file ppat.1007252.s005.tif]

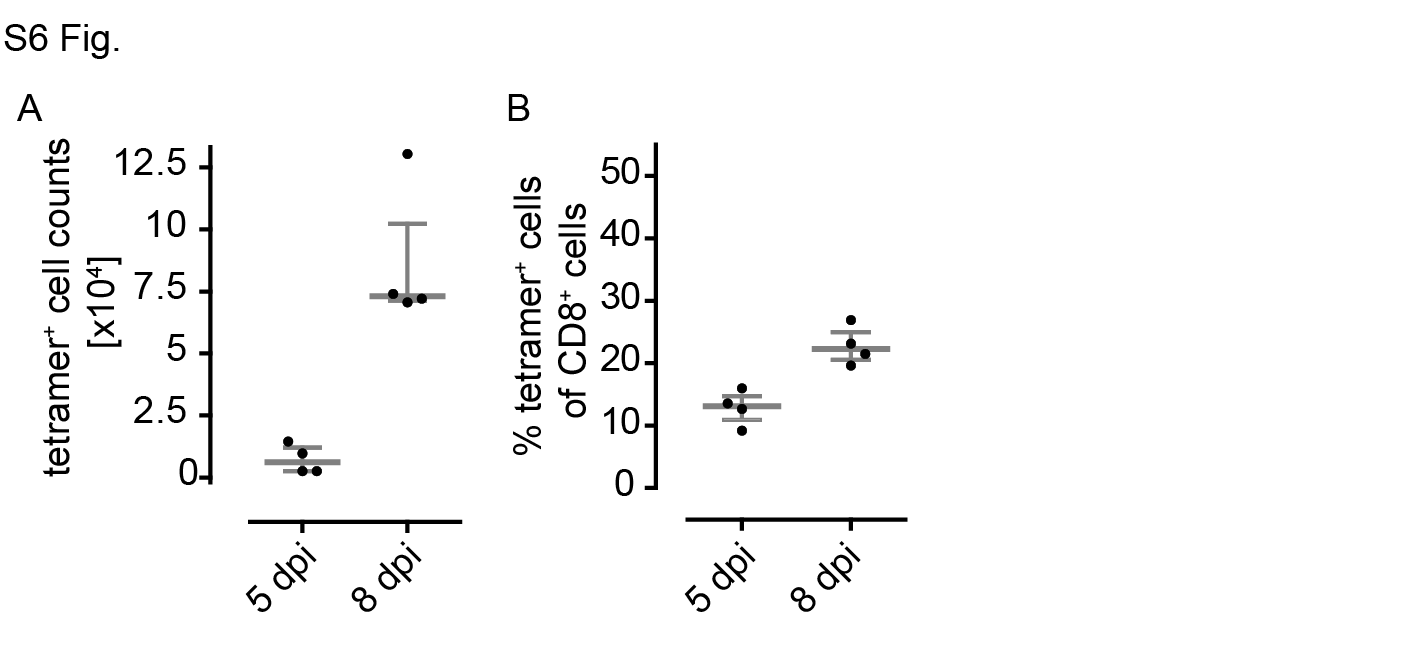

Supplement: S6 Fig — Animals were infected with 106 PFU MCMV-3D i.n. and lungs were analyzed at 5 and 8 dpi. For FACS analysis cells were stained with a mixture of three different tetramers (M45, m139, and M38). (A) Number of tetramer+ cells per lung and (B) frequency of tetramer+ cells of CD8+ T cells. Data points represent animals; median + interquartile range (grey); data from 2 experiments shown. (TIF) [file ppat.1007252.s006.tif]

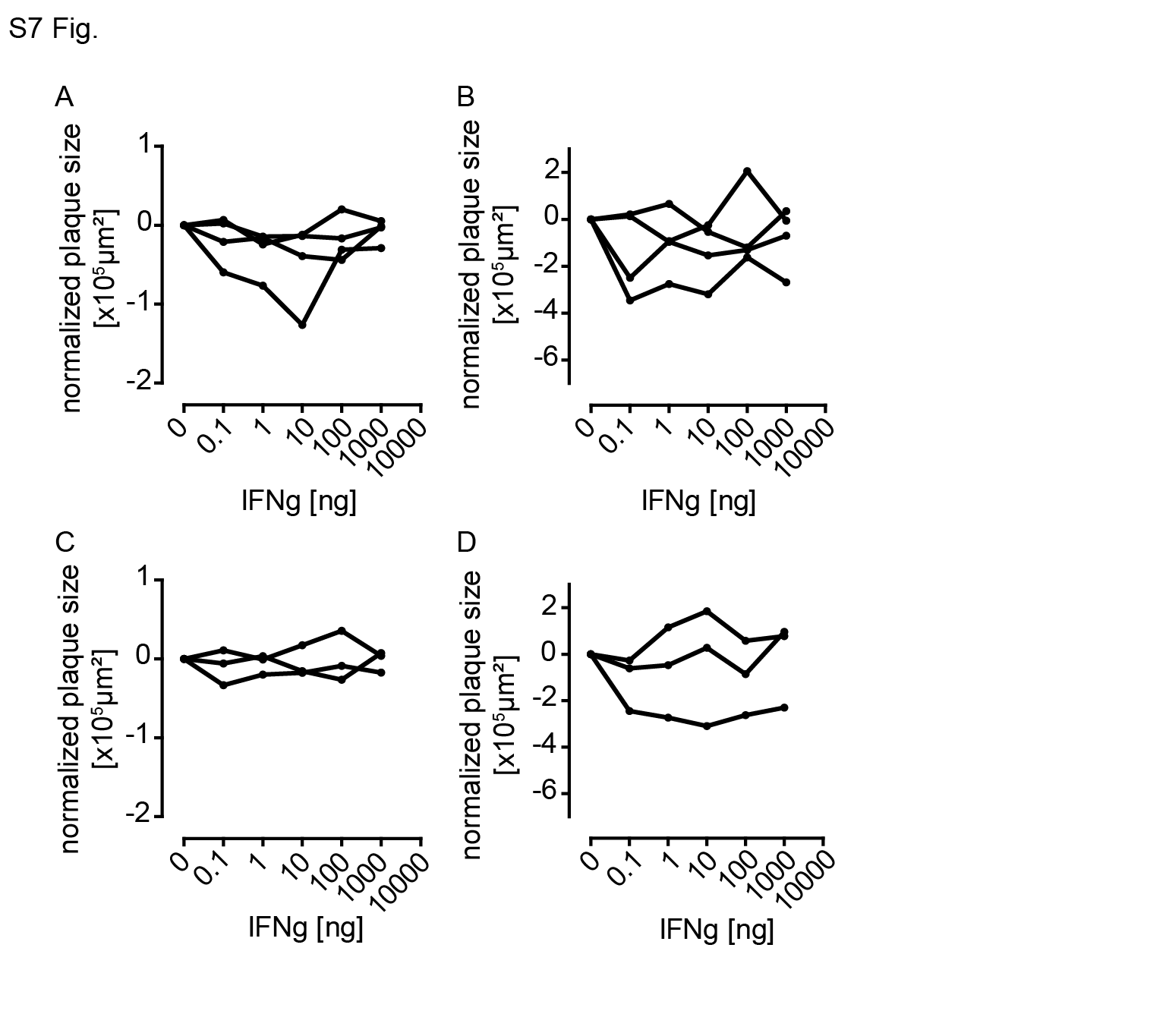

Supplement: S7 Fig — Primary lung stromal cells of Ifngr1-/- mice were cultured in the (A+B) presence or (C+D) absence of lung-resident CD45+ hematopoietic cells. Confluent cells were infected with 20 PFU MCMV-3D per well. After infection, carboxy-methyl-cellulose and various concentration of recombinant mouse IFNγ was added. Quantification of plaque sizes (A+C) 4 dpi and (B+D) 8 dpi after treatment with IFNγ as indicated. Mean of plaque sizes per well in dependence on the amount of IFNγ added (curves depict single experiments normalized to wells not treated with IFNγ). Data from 3–4 independent experiments are shown. (TIF) [file ppat.1007252.s007.tif]
